# Prevalence of Injection-Related Bacterial and Fungal Infection Among People Who Inject Drugs: A Systematic Review and Meta-analysis

**DOI:** 10.1093/ofid/ofaf108

**Published:** 2025-02-24

**Authors:** Alice Wheeler, Jeffrey Masters, Alyssa Pradhan, Jess Monineath Roth, Louisa Degenhardt, Gregory J Dore, Gail V Matthews, Evan B Cunningham, Amy Peacock, Samantha Colledge-Frisby, Jason Grebely, Behzad Hajarizadeh, Marianne Martinello

**Affiliations:** The Kirby Institute, UNSW Sydney, Sydney, New South Wales, Australia; The Kirby Institute, UNSW Sydney, Sydney, New South Wales, Australia; Department of Infectious Diseases, Royal Prince Alfred Hospital, Sydney, New South Wales, Australia; Department of Infectious Diseases, Westmead Hospital, Sydney, New South Wales, Australia; The Kirby Institute, UNSW Sydney, Sydney, New South Wales, Australia; National Drug and Alcohol Research Centre, UNSW Sydney, Sydney, New South Wales, Australia; The Kirby Institute, UNSW Sydney, Sydney, New South Wales, Australia; Department of Infectious Diseases, St Vincent's Hospital, Sydney, New South Wales, Australia; The Kirby Institute, UNSW Sydney, Sydney, New South Wales, Australia; Department of Infectious Diseases, St Vincent's Hospital, Sydney, New South Wales, Australia; The Kirby Institute, UNSW Sydney, Sydney, New South Wales, Australia; National Drug and Alcohol Research Centre, UNSW Sydney, Sydney, New South Wales, Australia; National Drug and Alcohol Research Centre, UNSW Sydney, Sydney, New South Wales, Australia; National Drug Research Institute, Curtin University, Perth, Western Australia, Australia; Burnet Institute, Melbourne, Victoria, Australia; The Kirby Institute, UNSW Sydney, Sydney, New South Wales, Australia; The Kirby Institute, UNSW Sydney, Sydney, New South Wales, Australia; The Kirby Institute, UNSW Sydney, Sydney, New South Wales, Australia; Department of Infectious Diseases, Prince of Wales Hospital, Sydney, New South Wales, Australia

**Keywords:** injecting drug use, injection-related infection, skin and soft tissue infection, harm reduction, prevalence

## Abstract

**Background:**

Despite the increasing burden of injection-related bacterial and fungal infections, there has been no recent synthesis of their epidemiology. We performed a systematic review and meta-analysis evaluating the prevalence and incidence of injection-related infections among people who inject drugs.

**Methods:**

We searched EMBASE, MEDLINE, Web of Science, and PsycINFO for articles published since 1 January 2010. Eligible studies assessed the prevalence or incidence of ≥1 injection-related infection among people who recently injected drugs. Random-effects meta-analysis was used to calculate pooled estimates of infection prevalence, according to infection type and prevalence period.

**Results:**

Of 8097 articles identified, 87 were eligible for inclusion (prevalence, 78; incidence, 9). Data were available for 25 countries, including 10 low- or middle-income countries. The prevalence of skin and soft-tissue infections (including skin abscess and cellulitis) was 13% in the past month (95% confidence interval [CI], 9%–19% [11 studies]), 30% in the past 3–12 months (23%–37% [23 studies]), and 47% across the lifetime (29%–66% [7 studies]). The prevalence of endocarditis was 2% in the past month (95% CI, 1%–3% [4 studies]), 2% in the past 3–12 months (2%–3% [5 studies]), and 6% across the lifetime (3%–10% [8 studies]). Prevalence of sepsis and/or bloodstream infection was 1% in the past month (95% CI, 1%–2% [2 studies]), 7% in the past 3–12 months (4%–13% [3 studies]), and 8% across the lifetime (3%–19% [5 studies]).

**Conclusions:**

Injection-related infections are a common complication of injecting drug use. Interventions to reduce their occurrence and associated disease burden are needed.

## Teaser

Injection-related infections are common among people who inject drugs, with prevalence varying by country and coverage of harm reduction services. Enhanced measures to prevent and manage injection-related infections are required to reduce morbidity and mortality rates among this population.

## Graphical Abstract

**Graphical Abstract:**
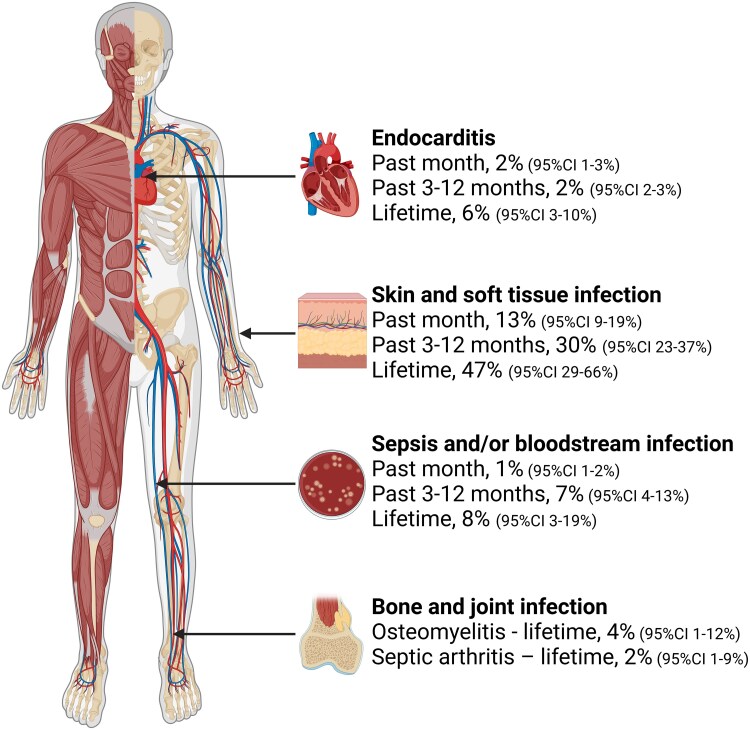
Created in BioRender. Martinello, M. (2025) https://BioRender.com/x25r557.

Bacterial and fungal infections acquired through exposure to pathogens in the injecting environment (hereafter referred to as *injection-related infection*) are a potential complication of injecting drug use [1–3]. The most common are localized infections of the skin and soft tissues (eg, abscess and cellulitis) that arise from introduction of microorganisms (ie, bacteria or fungi) into the skin and surrounding tissues when injecting [1, 2, 4]. Severe and systemic infections, such as endocarditis, bloodstream infection, osteomyelitis, and septic arthritis, can also occur, either via direct introduction of bacteria or fungi into the bloodstream and subsequent dissemination through the body or as a consequence of untreated or inadequately managed localized infection [2, 3]. Hospitalization of people who inject drugs (PWID) for the treatment of such infections is common and increasing in several countries, which has led to increased expenditure for healthcare systems [5–11]. At the individual level, injection-related infection can carry significant risk of severe disability and premature mortality and may require prolonged hospitalization with costly and invasive surgical procedures [12–14].

Existing reviews of injection-related infection are restricted to narrative or qualitative reviews lacking meta-analysis of available data [1, 15], are limited by a narrow focus on a single disease type (eg, skin and soft-tissue infection) [16], and feature small numbers of included studies, most published before the current decade [1, 16]. Updated and comprehensive reviews of infection prevalence, incidence, and associated risk factors are imperative to monitor the burden of injection-related infection among PWID and identify subpopulations at heightened risk of infection. To address this gap in the literature, we conducted a systematic review and meta-analysis to evaluate the prevalence and incidence of injection-related infection among PWID.

## METHODS

This systematic review and meta-analysis is reported in accordance with PRISMA [17] and GATHER [18] statements (Supplementary Tables 1 and 2). The study protocol is registered with PROSPERO (CRD42022360163).

### Search Strategy and Selection Criteria

We systematically searched the following bibliographic databases for relevant peer-reviewed literature published from 1 January 2010 onward: EMBASE, MEDLINE (PubMed), Web of Science, and PsycINFO. Search strings were developed in consultation with a specialist librarian and included combinations of search terms related to injecting drug use, opioid agonist treatment, and injection-related infection. Full details of each search strategy are provided in the Appendix (Supplementary Table 3). Initial searches were conducted by A. W. on 12 October 2022 and updated on 12 November 2024. No language restrictions were used. Relevant review articles retrieved in the search were retained and hand-searched for eligible cited literature. Relevant study protocols were also retained and the authors contacted to enquire about availability of data. All first and senior authors of included studies were contacted via email to obtain additional data and clarify study details if necessary.

Searches were supplemented with articles obtained through a broader systematic review conducted previously by the study authors, which evaluated the prevalence of injecting drug use and sociodemographic characteristics of PWID (CRD42020173337) [4]. Briefly, in 2020–2022, peer-reviewed databases (MEDLINE, Embase, and PsycINFO) were searched without language restrictions for articles with estimates of injecting prevalence and/or prevalence of ≥1 of the following among PWID: sociodemographic and injecting risk characteristics, use of harm reduction services, blood-borne viral infection, and injection-related bacterial or fungal infection. An iterative search of nonindexed government and nongovernment reports (gray literature) was also performed using an established list of online websites identified as having potential relevance to the review [19]. Peer-reviewed and gray literature articles included in the broader review and reporting an estimate of injection-related infection prevalence were assessed for eligibility in the current review, if not already included.

Studies were included if they had a study population comprising people who injected drugs and/or received opioid agonist treatment in the past 12 months (ie, recently) and if they reported the number or proportion of the study sample with a current or previous injection-related infection (prevalence) or the rate of new cases of infection across a specified period (incidence). Study populations comprising people who recently received opioid agonist treatment were included given the high prevalence of injecting drug use in this population [20]. Acceptable methods of ascertaining recent injecting drug use and/or receipt of opioid agonist treatment included participant self-report, review of clinical records, medical assessment by a clinician, or a combination of these methods. Included studies were required to report the prevalence or incidence of a specified infection or combination of infections attributable to injecting drug use, with infection history assessed via participant self-report, review of clinical records (for diagnoses recorded using *International Classification of Diseases* codes or other methods), and/or medical assessment. Studies of incidence were included only if the rate of infection, including person-years of follow-up, was reported or could be calculated using available data.

Studies were excluded if the sample comprised people recruited from an inpatient hospital unit or people exclusively injecting image- and performance-enhancing drugs or if the presence of injection-related infection was a criterion for study participation/eligibility. Studies were also excluded if the outcome reported was a health condition, or combination of health conditions, in which the presence of infection and/or the attribution of injecting drug use were ambiguous (eg, skin ulcer). In the case of multiple reports or publications of a single study, we cited the article providing the most comprehensive and relevant data from the study population.

### Data Analysis

Identified articles were deduplicated in EndNote X9 and imported into COVIDence for title, abstract, and full-text screening. All articles were screened by 2 independent reviewers (including A. W., J. M., A. Pradhan, J. G., B. H., or M. M.) with conflicts resolved via discussion with a third reviewer (B. H. or M. M.). Data from eligible studies were extracted into a custom-built Microsoft Excel database by A. W., J. M., A. Pradhan, J. M. R., or B. H. and double-checked for accuracy by a second team member (A. W., J. M., A. Pradhan, or J. M. R.). Extracted data items included the study setting, participant recruitment method and inclusion criteria, basic participant characteristics (eg, age, sex, and human immunodeficiency virus status), method of assessing participant infection history (eg, self-report or clinical record/assessment), number or proportion of the study population with an injection-related infection (prevalence), and the rate of new cases of infection, including person-years of follow-up (incidence). A complete list of extracted data items can be found in the Appendix (Supplementary Table 4).

The risk of bias in included studies was assessed using modified versions of the Joanna Briggs Institute Critical Appraisal Checklist for prevalence studies [21] if infection prevalence was reported or the Newcastle-Ottawa Scale for cohort studies [22] if infection incidence was reported. Modifications to each tool are detailed in the Appendix (Supplementary Tables 5–8). The risk of bias assessments were performed independently by 2 team members (including A. W., J. M., A. Pradhan, J. M. R., or B. H.).

Random-effects meta-analysis was used to calculate pooled estimates of infection prevalence, according to infection type and prevalence period. Infection types were established using definitions provided by individual studies and included the following: skin and soft-tissue infection, skin abscess, cellulitis, endocarditis, bloodstream infection and/or sepsis, osteomyelitis, and septic arthritis. Pooled prevalence estimates were calculated for infection types only where data were available from ≥2 unique studies. For skin and soft-tissue infection, pooled estimates were derived independently of estimates for skin abscess and cellulitis, using only studies that reported skin and soft-tissue infection as a composite outcome.

Where sufficient data were available, we also calculated pooled estimates of infection prevalence stratified by (1) country, (2) country income level (high-income vs low-middle-income countries), and (3) country-level coverage of harm reduction services for PWID (high vs other coverage levels). Country income level was determined using the World Bank country income classification [23]. Harm reduction categorization was informed by previous estimates of the coverage of needle-syringe programs and opioid agonist treatment for PWID [24]. All analyses were performed using Stata software, version 18.0.

## RESULTS

**Figure 1.**
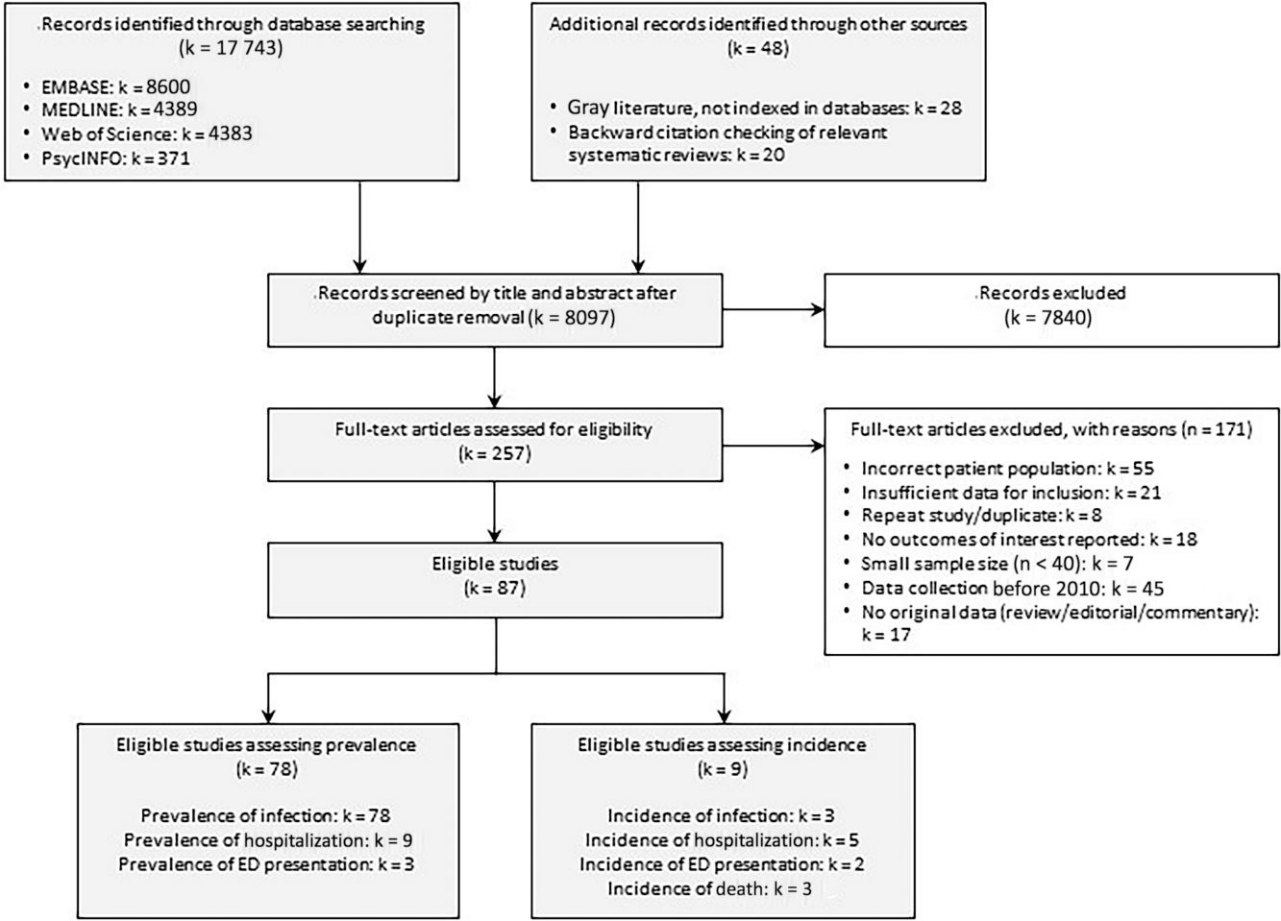
Flow diagram detailing the search and screening process (PRISMA flow diagram). Abbreviations: ED, emergency department; k represents number of studies, and n, number of participants.

We identified 17 743 peer-reviewed articles from bibliographic databases and 48 additional articles from other sources, resulting in 8097 unique articles for screening after duplicate removal (Figure 1). Of these, 87 were eligible for inclusion in the review (78 and 9 articles assessing the prevalence and incidence of injection-related infection, respectively; Supplementary Tables 9 and 10), and 77 were eligible for inclusion in meta-analyses of prevalence.

Of 78 studies assessing the prevalence of injection-related infection, the majority (97%) included a study population defined as PWID, with only 2 studies (3%) conducted among a population of people receiving opioid agonist treatment (Table 1). Most studies relied on participant self-report to ascertain both injecting status (79%) and infection history (89%). Review of clinical records and/or clinical assessment was used to determine infection outcomes in 6 (8%) studies, while 2 used *International Classification of Diseases* codes (3%). Most prevalence studies (88%) were conducted in high-income countries, with common settings of participant recruitment including needle-syringe programs (32%), other harm reduction or drug treatment services (23%), and mixed settings (26%) (Table 1). Country-level harm reduction provision among PWID varied, with 22% and 49% of studies performed in countries with high-coverage needle-syringe programs or high-coverage opioid agonist treatment, respectively (Table 1).

**Table 1.** Characteristics of Included Studies Assessing the Prevalence of Injection-Related Infection Among People Who Inject Drugs

Characteristic	Studies, No. (%)	Study Participants, No. (%)
Total	78 (100)	950 301 (100)
Study design		
Cross-sectional study	63 (81)	46 729 (5)
Retrospective cohort study	6 (8)	901 157 (95)
Prospective cohort study	4 (5)	1753 (<1)
Clinical trial	5 (6)	662 (<1)
Study setting		
Needle-syringe program	25 (32)	17 786 (2)
Opioid agonist treatment clinic	3 (4)	2974 (<1)
Other harm reduction or drug treatment service	18 (23)	11 172 (1)
Community-based/street outreach	11 (14)	3836 (<1)
Primary care/general practice	1 (1)	216 (<1)
Multiple settings	20 (26)	914 317 (96)
No. of study sites		
Single site	9 (12)	825 (<1)
Multiple sites	69 (88)	949 415 (100)
Population		
People who inject drugs	76 (97)	893 550 (94)
People receiving opioid agonist treatment	2 (3)	56 751 (6)
Method of defining study population		
Self-reported IDU or OAT	62 (79)	46 022 (5)
Clinical record/assessment of IDU or OAT	7 (9)	2253 (<1)
*ICD* codes for drug use	2 (3)	844 458 (89)
Other or unspecified methods	7 (9)	57 568 (6)
Proportion of study population on OAT		
≥70%	7 (9)	57 998 (6)
≥30% and <70%	19 (24)	15 289 (2)
<30%	5 (6)	3177 (<1)
Unspecified	47 (60)	873 810 (92)
Outcome (infection) reported^a^		
Skin and soft-tissue infection NOS	39 (50)	29 929 (3)
Abscess	31 (40)	12 043 (1)
Cellulitis	6 (8)	2711 (<1)
Endocarditis	17 (22)	67 454 (7)
Bloodstream infection	10 (13)	3774 (<1)
Bone or joint infection	7 (9)	113 964 (12)
Any injection-related infection	20 (26)	14 944 (2)
Multiple injection-related infections	6 (8)	902 062 (95)
Other injection-related infection	10 (13)	7575 (1)
Method of outcome (infection) measurement		
Self-reported	70 (89)	48 837 (5)
Clinical record audit and/or clinical assessment	6 (8)	56 911 (6)
*ICD* codes for infection	2 (3)	844 458 (89)
Outcome (infection) time frame^a^		
Lifetime/ever	47 (60)	1 084 436 (>100)
Past 12 mo	28 (36)	24 990 (3)
Past 6 mo	15 (19)	9022 (1)
Past 3 mo	3 (4)	1108 (<1)
Past mo	37 (47)	25 971 (3)
Current	5 (6)	1083 (<1)
Country income status		
High income	69 (88)	945 756 (100)
Low or middle income	9 (12)	4545 (<1)
Country harm reduction status^b^		
High-coverage needle-syringe program	17 (22)	69 253 (7)
High-coverage OAT	38 (49)	86 856 (9)
High-coverage needle-syringe program + OAT	18 (23)	69 314 (7)

Abbreviations: *ICD, International Classification of Diseases*; IDU, injecting drug use; NOS, not otherwise specified; OAT, opioid agonist treatment.

^a^As studies may have assessed >1 infection type and/or over different time periods, the total numbers of studies and participants are greater than 78 (100%) and 950 301 (100%), respectively.

^b^Harm reduction status categorized according to Colledge-Frisby et al [24].

All studies evaluating the prevalence of infection were at risk of selection bias, largely due to the use of nonprobability sampling techniques (eg, convenience sampling) for participant recruitment and inclusion/exclusion criteria inadequate for generating a sample that closely represented the broader population of interest (PWID) (Supplementary Table 6). The majority of prevalence studies (87%; k = 68) were also at risk of misclassification bias due to the use of nonobjective methods of infection assessment, particularly participant self-report (Supplementary Table 6).

**Figure 2.**
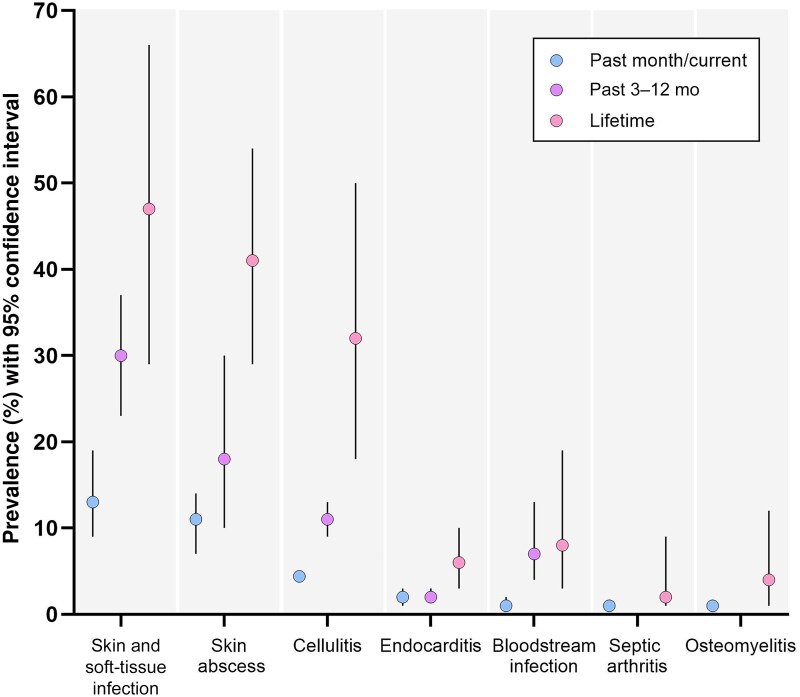
Pooled estimates of the prevalence of injection-related infection among people who inject drugs, by infection type and prevalence period. Abbreviation: CI, confidence interval.

**Figure 3.**
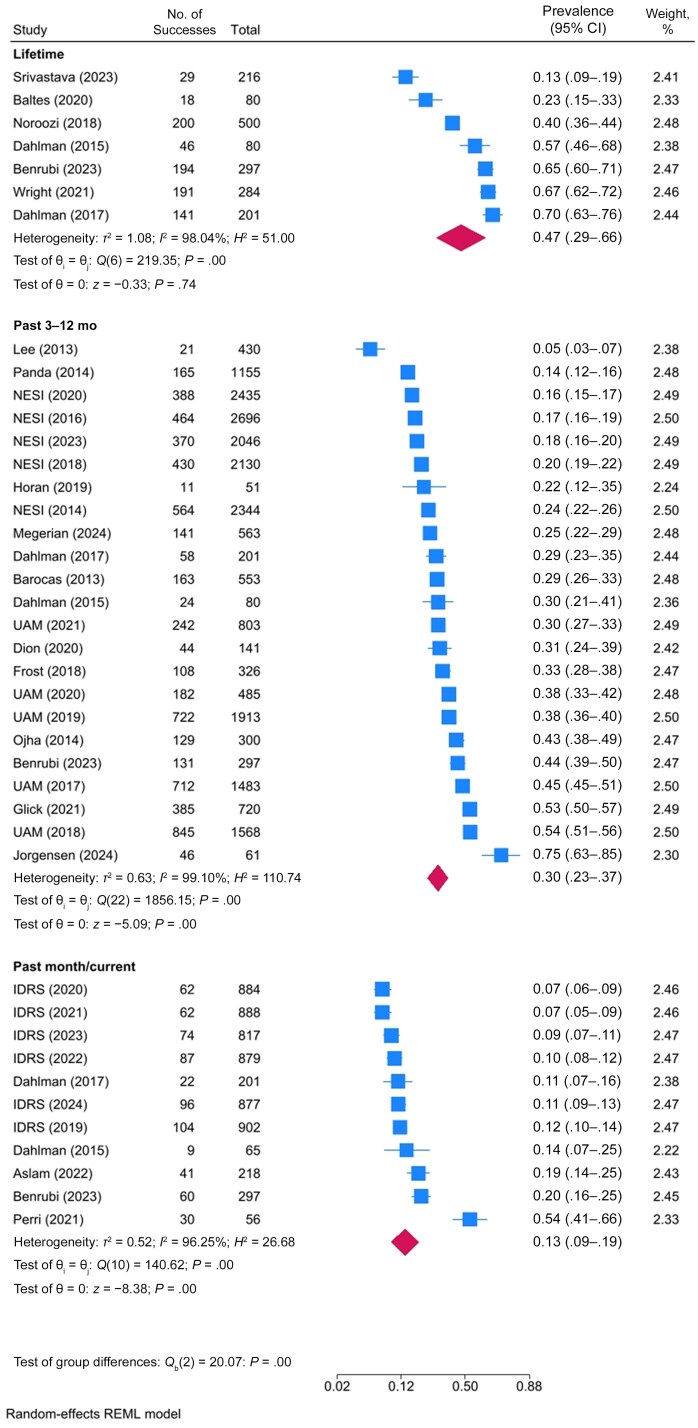
Forest plot of the prevalence of skin and soft-tissue infections among people who inject drugs, by prevalence period. Abbreviations: IDRS, Illicit Drug Reporting System; NESI, Needle Exchange Surveillance Initiative; REML, restricted maximum likelihood; UAM, Unlinked Anonymous Monitoring Survey. Listed studies are referenced in the supplementary reference list.

Thirty-six studies reported an estimate of the prevalence of skin and soft-tissue infection among PWID (Supplementary Table 11). The pooled prevalence of skin and soft-tissue infections was 13% in the past month (95% confidence interval [CI], 9%–19% [11 studies]), 30% in the past 3–12 months (23%–37% [23 studies]), and 47% across the lifetime (29%–66% [7 studies]) (Table 2 and Figures 2 and 3).

**Table 2.** Pooled Estimates of the Prevalence of Injection-Related Infection Among People Who Inject Drugs, by Infection Type and Prevalence Period^a^

Type of Infection	Current or Past Month			Past 3–12 mo			Lifetime		
	Studies, No.	Participants, No.	Estimated Prevalence (95% CI), %	studies, No.	Participants, No.	Estimated Prevalence (95% CI), %	Studies, No.	Participants, No.	Estimated Prevalence (95% CI), %
Skin and soft-tissue infection NOS	11	6084	13 (9–19)	23	22 781	30 (23–37)	7	1658	47 (29–66)
Abscess	6	1173	11 (7–14)	10	4867	18 (10–30)	15	6003	41 (29–54)
Cellulitis	1	…	…	2	969	11 (9–13)	3	1697	32 (18–50)
Endocarditis	4	3546	2 (1–3)	5	3582	2 (2–3)	8	60 326	6 (3–10)
Bloodstream infection	2	947	1 (1–2)	3	1587	7 (4–13)	5	1240	8 (3–19)
Osteomyelitis	1	…	…	0	…	…	3	56 140	4 (1–12)
Septic arthritis	1	…	…	0	…	…	3	56 140	2(1–9)

Abbreviations: CI, confidence interval; NOS, not otherwise specified.

^a^Pooled estimates were calculated for infection types when ≥2 included studies were available. References and summary data for included studies are available in the Appendix, including Supplementary Table 11 (skin and soft-tissue infection NOS), Supplementary Table 12 (abscess), Supplementary Table 13 (cellulitis), Supplementary Table 18 (endocarditis), Supplementary Table 19 (bloodstream infection and/or sepsis), Supplementary Table 20 (osteomyelitis), and Supplementary Table 21 (septic arthritis).

**Figure 4.**
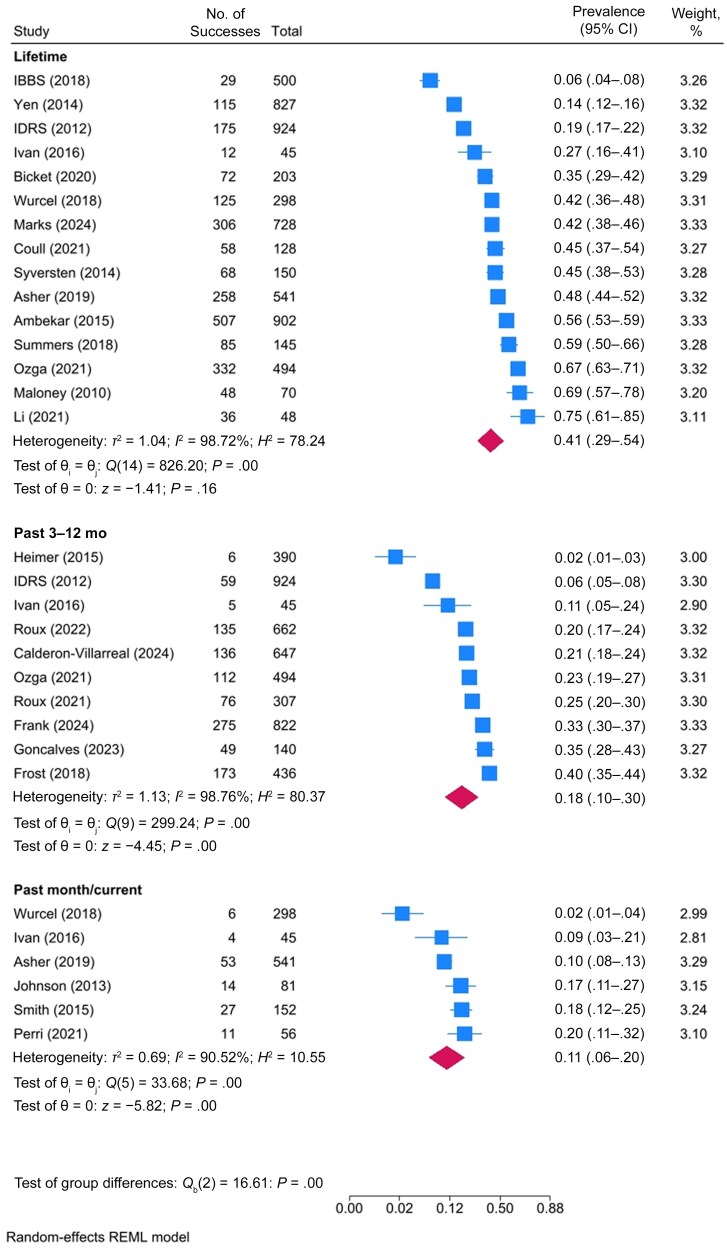
Forest plot of the prevalence of abscesses among people who inject drugs, by prevalence period. Abbreviations: IBBS, Integrated Bio-Behavioural Survey; IDRS, Illicit Drug Reporting System; REML, restricted maximum likelihood. Listed studies are referenced in the supplementary reference list.

Twenty-five studies reported the prevalence of a specified skin and soft-tissue infection, specifically abscess or cellulitis, among PWID (Supplementary Tables 12 and 13). Among 25 studies reporting the prevalence of skin abscess, the pooled prevalence was 11% in the past month (95% CI, 7%–14% [6 studies]), 18% in the past 3–12 months (10%–30% [10 studies]), and 41% across the lifetime (29%–54% [15 studies]) (Table 2 and Figures 2 and 4). Among 3 studies reporting the prevalence of cellulitis among PWID, the pooled prevalence of cellulitis was 11% in the past 3–12 months (95% CI, 9%–13% [2 studies]) and 32% across the lifetime (18%–50% [3 studies]) (Table 2, Figure 2, and Supplementary Figure 1).

Prevalence estimates for skin and soft-tissue infection were lower in countries with high harm reduction coverage. Prevalence of skin and soft-tissue infection in the last 12 months was 9% (95% CI, 8%–11% [6 studies]) in countries with high-coverage needle-syringe programs, compared with 28% (23%–35% [28 studies]) in countries without (Supplementary Table 14 and Supplementary Figure 2). The prevalence of skin and soft-tissue infection in the last 12 months was 17% (95% CI, 11%–24% [15 studies]) in countries with high-coverage opioid agonist therapy, compared with 30% (23%–38% [19 studies]) in countries without (Supplementary Table 14 and Supplementary Figure 2). There was no difference in prevalence estimates for skin and soft-tissue infection by country income status (Supplementary Table 15), but there were differences between countries (Supplementary Table 16 and Supplementary Figure 3). Only 6 studies evaluated the prevalence of hospitalization for skin and soft-tissue infection, either as a composite (any skin and soft-tissue infection) or a single (skin abscess or cellulitis) infection type, with estimates ranging from 1% in the past 3 months [25] to 20% in the past 12 months [26] (Supplementary Table 17).

**Figure 5.**
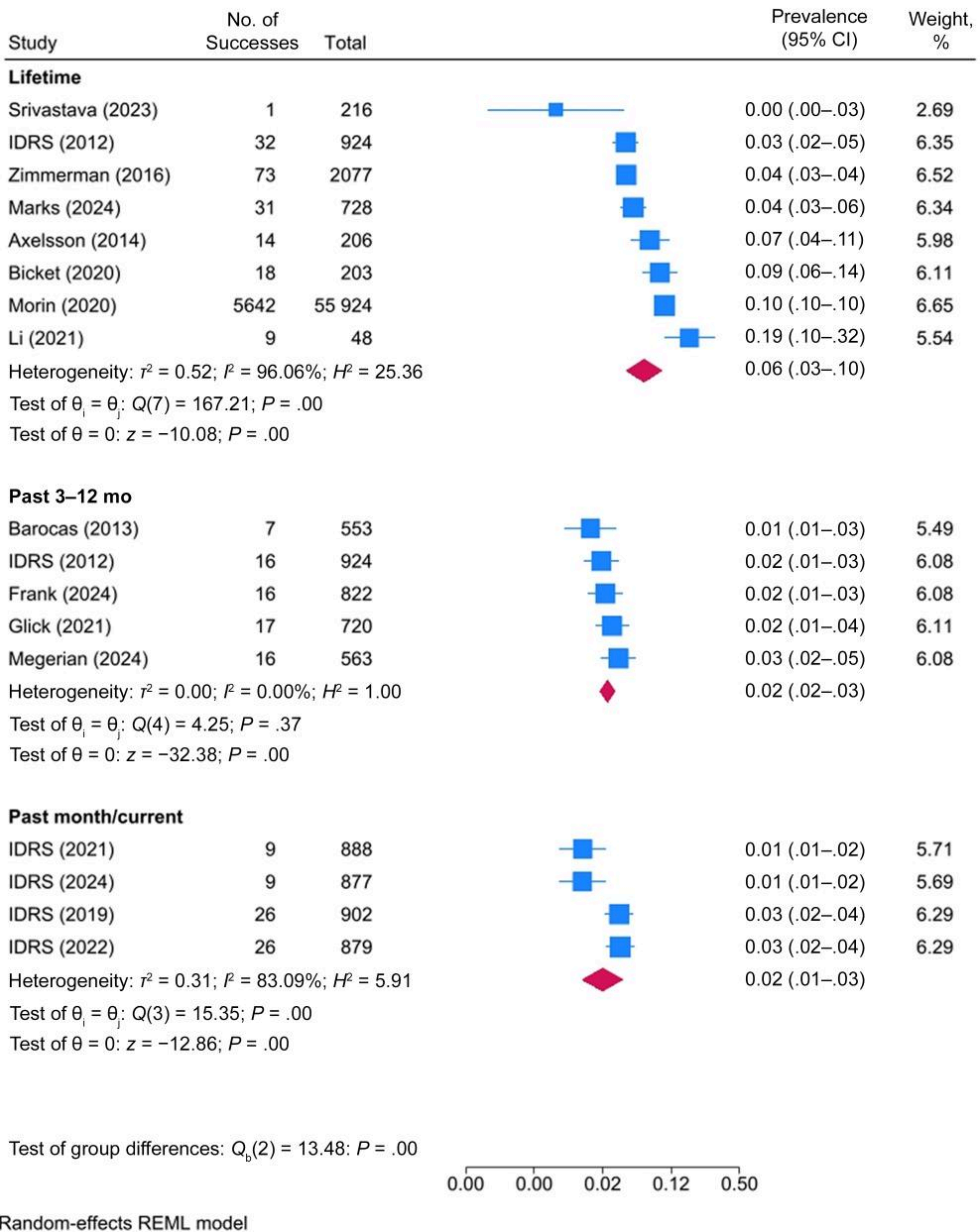
Forest plot of the prevalence of endocarditis in people who inject drugs, by prevalence period. Abbreviations: IDRS, Illicit Drug Reporting System; REML, restricted maximum likelihood. Listed studies are referenced in the supplementary reference list.

Sixteen studies reported an estimate of the prevalence of endocarditis among PWID (Supplementary Table 18). The pooled prevalence of endocarditis was 2% in the past month (95% CI, 1%–3% [4 studies]), 2% in the past 3–12 months (2%–3% [5 studies]), and 6% across the lifetime (3%–10% [8 studies]) (Table 2 and Figures 2 and 5).

Ten studies reported an estimate of the prevalence of sepsis and/or bloodstream infection among PWID (Supplementary Table 19). The pooled prevalence of sepsis and/or bloodstream infection was 1% in the past month (95% CI, 1%–2% [2 studies]), 7% in the past 3–12 months (4%–13% [3 studies]), and 8% across the lifetime (3%–19% [5 studies]) (Table 2, Figure 2, and Supplementary Figure 4).

Four studies reported an estimate of the prevalence of bone or joint infection (osteomyelitis or septic arthritis) among PWID (Supplementary Tables 20 and 21). Pooled lifetime prevalence estimates were 4% for osteomyelitis (95% CI, 1%–12% [3 studies]) and 2% for septic arthritis (1%–9% [3 studies]) (Table 2 and Figure 2).

Studies reporting the prevalence of other outcomes are listed in the Appendix (Supplementary Tables 22–24). Nine studies evaluating the incidence of injection-related infection among PWID were eligible for inclusion but could not be meta-analyzed due to heterogeneity of outcomes (Supplementary Tables 8 and 10).

## DISCUSSION

With 14.8 million people estimated to inject drugs globally in 2021 [4], the individual- and population-level burdens of injection-related infection are considerable. Skin and soft-tissue infection (including abscess and cellulitis) were the most prevalent injection-related infection (past month, 13%; past 3–12 months, 30%; lifetime, 47%). More severe invasive infection (for which hospitalization and multidisciplinary management is often required) occurred in up to 7% within the last year, including sepsis and/or bloodstream infection (past month, 1%; past 3–12 months, 7%; lifetime, 8%), endocarditis (past month, 2%; past 3–12 months, 2%; lifetime, 6%), and bone and joint infection, including osteomyelitis (lifetime, 4%) and septic arthritis (lifetime, 2%).

Injection-related infections are a common complication of injecting drug use, but synthesis of data had been limited to date. With a broad search (including published and gray literature), we identified sufficient studies to calculate pooled prevalence estimates of specific injection-related infections at different periods in time, expanding on previous literature. Our estimate of the past-year prevalence of skin and soft-tissue infection was similar to another recent global meta-analysis [4]. We were also able to provide estimates of prevalence for other serious injection-related infections, including endocarditis, bloodstream infection/sepsis, osteomyelitis, and septic arthritis. However, we were unable to calculate estimates of infection-specific incidence or hospitalization, given insufficient data. Most studies relied on self-report of injecting drug use and infection which may introduce concerns regarding misclassification bias. Supporting validity of self-report, similar prevalence estimates were obtained from studies that used other methods of outcome ascertainment, including clinical review and audit.

The prevalence of injection-related infections among PWID varied between countries and across regions. Individual factors may increase infection risk, including skin colonization (particularly with methicillin-resistant *Staphylococcus aureus*) [27] and housing instability [28]. Specific drug use behaviors may also contribute, including frequency of injection [29–31], site of injection [29, 32], mode of injection [31, 33], type of drug injected [31, 34, 35], and use of sterile injecting equipment and paraphernalia [28, 30]; for example, increased risk of skin and soft-tissue infection has been associated with daily (or more often) injecting, injection into the neck or lower limbs, subcutaneous (“skin popping”) or intramuscular injection, use of black tar heroin, and reuse of one's own needles. While individual sociodemographic factors and drug use patterns are important, equitable healthcare and harm reduction access are vital. With safer injecting practices associated with lower risk of injection-related infection [36], access to harm reduction and drug treatment programs are essential for PWID to reduce risk of all drug-related harms, including overdose and blood-borne virus transmission.

Importantly, we showed that the prevalence of skin and soft-tissue infection was lower in countries with high harm reduction coverage. Although there is evidence of opioid agonist treatment and/or needle-syringe programs in >90 countries, global coverage has remained suboptimal; only 5 countries (including 2% of the population of PWID) provide high coverage of both services, with most countries well below international service delivery targets [24].

Over the last decade, an increase in health service use and hospitalization for injection-related infections has been reported in some high- and low-middle-income countries [5–11]. Treatment of severe and/or invasive injection-related infection often requires prolonged hospitalization, intravenous antibiotics, and multidisciplinary management. Although injection-related infection is a source of considerable individual morbidity and healthcare expenditure, our evaluation of the burden of hospitalization due to such infection was limited; given the lack of data, we were unable to calculate pooled estimates for individual infection types.

The prevalence estimates we derived for severe and/or systemic infection (eg, endocarditis, bloodstream infection) are likely to reflect the prevalence of hospitalization as diagnosis and management will predominately have occurred in a hospital setting. However, the prevalence estimates we derived for skin and soft-tissue infection are likely to differ from the prevalence of hospitalization for these infections, as skin and soft-tissue infection can range in severity (from mild to life-threatening), with diagnosis and management of skin and soft-tissue infection without systemic features often occurring in outpatient or community settings. In addition, a substantial proportion of PWID have reported self-management of skin and soft-tissue infection; for example, of 1289 PWID in a US sample, 63% reported ever having had an injection-related infection, 29% had drained abscesses without seeking healthcare, and 23% had acquired antibiotics without medical attention [37].

PWID face unique challenges during health service interactions (stigma, withdrawal, pain, mental health, competing social priorities). A range of management options (including alternative antibiotic delivery) to alleviate prolonged hospitalization are available and can be tailored in a person-centered manner; however, PWID are often excluded from novel models of care. Further data on hospitalization and health service engagement for injection-related infection among community-based cohorts are required to assess burden and improve care delivery. Implementation of stigma-reduction interventions in healthcare settings could be particularly beneficial, given that stigmatizing experiences in such contexts is a key factor contributing to avoidance or delay of seeking medical care among PWID [38].

Additional considerations of available data include geographic restriction, bias in data sources, varied definitions of infection, use of composite end points, and sampling methods. Studies of prevalence of injection-related infection have largely been performed in high-income countries among PWID who are in contact with health services (including needle-syringe programs and drug health clinics), which may affect generalizability. All prevalence estimates for severe injection-related infections (endocarditis, bloodstream infection, osteomyelitis, and septic arthritis) were from high-income countries, highlighting a key data gap. We were unable to calculate pooled prevalence estimates by sex as most studies did not report disaggregated data; given suggestions of sex-based differences in infection risk [26, 39], this should be considered and reported in future work.

Definitions of injection-related infection varied between studies, with some reporting nonspecific composite outcomes that combined various infection types and severity; we excluded studies that reported only on “injection-related infection” overall from meta-analyses. Use of clinical or billing codes may underestimate prevalence of less severe infections, with reports of self-treatment of localized skin and soft-tissue infection without seeking medical attention [37, 40]. Importantly, stigma associated with injecting drug use continues to impair evaluation substantially [38].

Despite advances in the prevention and treatment of infectious diseases, significant gaps persist in optimal and equitable health delivery to the most vulnerable. With >30% of PWID reporting injection-related infections in the last year, prevention and management must be enhanced to improve quality of life and reduce morbidity. Leveraging global momentum for blood-borne virus elimination offers an opportunity to implement proven and evaluate new strategies to improve health outcomes among PWID, addressing preventable infections associated with vulnerability and disadvantage.

## Supplementary Material

ofaf108_Supplementary_Data

## Supplementary Data

Supplementary materials are available at *Open Forum Infectious Diseases* online. Consisting of data provided by the authors to benefit the reader, the posted materials are not copyedited and are the sole responsibility of the authors, so questions or comments should be addressed to the corresponding author.
