# Supplementary material for: Prevalence of Injection-Related Bacterial and Fungal Infection Among People Who Inject Drugs: A Systematic Review and Meta-analysis
Source: Open Forum Infect Dis. 2025 Feb 24;12(4):ofaf108. doi: 10.1093/ofid/ofaf108 (PMC11952969; doi:10.1093/ofid/ofaf108)
Supplement: ofaf108_Supplementary_Data [file ofaf108_supplementary_data.docx]

**Supplementary materials for the article:**

**Prevalence of injecting-related bacterial and fungal infection among people who inject drugs: a systematic review and meta-analysis**

**Authors: Alice Wheeler^1^, Jeffrey Masters^1,2^, Alyssa Pradhan^3^, Jess Monineath Roth^1^, Louisa Degenhardt^4^, Gregory J Dore^1,5^, Gail V Matthews^1,5^, Evan B Cunningham^1^, Amy Peacock^4^, Samantha Colledge-Frisby^4,6,7^, Jason Grebely^1^, Behzad Hajarizadeh^1^*, Marianne Martinello^1,8^***

^1^ The Kirby Institute, UNSW Sydney, Sydney, New South Wales, Australia

^2^ Department of Infectious Diseases, Royal Prince Alfred Hospital, Sydney, New South Wales, Australia

^3^ Department of Infectious Diseases, Westmead Hospital, Sydney, New South Wales, Australia

^4^ National Drug and Alcohol Research Centre, UNSW Sydney, Sydney, New South Wales, Australia

^5^ Department of Infectious Diseases, St. Vincent’s Hospital, Sydney, New South Wales, Australia

^6^ National Drug Research Institute, Curtin University, Perth, Western Australia, Australia

^7^ Burnet Institute, Melbourne, Victoria, Australia

^8^ Department of Infectious Diseases, Prince of Wales Hospital, Sydney, New South Wales, Australia

*Joint senior author

**Supplementary table 1.** Preferred Reporting Items for Systematic Reviews and Meta-Analyses (PRISMA) checklist

| **Section and Topic** | **Item #** | **Checklist item** | **Location where item is reported** |
| --- | --- | --- | --- |
| **TITLE** | | |  |
| Title | 1 | Identify the report as a systematic review. | Pg. 1 |
| **ABSTRACT** | | |  |
| Abstract | 2 | See the PRISMA 2020 for Abstracts checklist. | Pg. 2 |
| **INTRODUCTION** | | |  |
| Rationale | 3 | Describe the rationale for the review in the context of existing knowledge. | Pg. 5 |
| Objectives | 4 | Provide an explicit statement of the objective(s) or question(s) the review addresses. | Pg. 5 |
| **METHODS** | | |  |
| Eligibility criteria | 5 | Specify the inclusion and exclusion criteria for the review and how studies were grouped for the syntheses. | Pg. 7 |
| Information sources | 6 | Specify all databases, registers, websites, organisations, reference lists and other sources searched or consulted to identify studies. Specify the date when each source was last searched or consulted. | Pg. 6-7 |
| Search strategy | 7 | Present the full search strategies for all databases, registers, and websites, including any filters and limits used. | Appendix pg. 6-8 |
| Selection process | 8 | Specify the methods used to decide whether a study met the inclusion criteria of the review, including how many reviewers screened each record and each report retrieved, whether they worked independently, and if applicable, details of automation tools used in the process. | Pg. 7-8 |
| Data collection process | 9 | Specify the methods used to collect data from reports, including how many reviewers collected data from each report, whether they worked independently, any processes for obtaining or confirming data from study investigators, and if applicable, details of automation tools used in the process. | Pg. 8 |
| Data items | 10a | List and define all outcomes for which data were sought. Specify whether all results that were compatible with each outcome domain in each study were sought (e.g., for all measures, time points, analyses), and if not, the methods used to decide which results to collect. | Pg. 8  Appendix pg. 9-11 |
|  | 10b | List and define all other variables for which data were sought (e.g., participant and intervention characteristics, funding sources). Describe any assumptions made about any missing or unclear information. | Pg. 8  Appendix pg. 9-11 |
| Study risk of bias assessment | 11 | Specify the methods used to assess risk of bias in the included studies, including details of the tool(s) used, how many reviewers assessed each study and whether they worked independently, and if applicable, details of automation tools used in the process. | Pg. 8  Appendix pg. 12-13, 18-19 |
| Effect measures | 12 | Specify for each outcome the effect measure(s) (e.g., risk ratio, mean difference) used in the synthesis or presentation of results. | Pg. 8-9 |
| Synthesis methods | 13a | Describe the processes used to decide which studies were eligible for each synthesis (e.g., tabulating the study intervention characteristics and comparing against the planned groups for each synthesis (item #5)). | Pg. 8-9 |
|  | 13b | Describe any methods required to prepare the data for presentation or synthesis, such as handling of missing summary statistics, or data conversions. | Pg. 8-9 |
|  | 13c | Describe any methods used to tabulate or visually display results of individual studies and syntheses. | Pg. 8-9 |
|  | 13d | Describe any methods used to synthesize results and provide a rationale for the choice(s). If meta-analysis was performed, describe the model(s), method(s) to identify the presence and extent of statistical heterogeneity, and software package(s) used. | Pg. 8-9 |
|  | 13e | Describe any methods used to explore possible causes of heterogeneity among study results (e.g., subgroup analysis, meta-regression). | NA |
|  | 13f | Describe any sensitivity analyses conducted to assess robustness of the synthesized results. | NA |
| Reporting bias assessment | 14 | Describe any methods used to assess risk of bias due to missing results in a synthesis (arising from reporting biases). | NA |
| Certainty assessment | 15 | Describe any methods used to assess certainty (or confidence) in the body of evidence for an outcome. | NA |
| **RESULTS** | | |  |
| Study selection | 16a | Describe the results of the search and selection process, from the number of records identified in the search to the number of studies included in the review, ideally using a flow diagram. | Pg. 10, 28 |
|  | 16b | Cite studies that might appear to meet the inclusion criteria, but which were excluded, and explain why they were excluded. | Pg. 28 |
| Study characteristics | 17 | Cite each included study and present its characteristics. | Appendix pg. 21-29, 54-58 |
| Risk of bias in studies | 18 | Present assessments of risk of bias for each included study. | Appendix pg. 14-17, 20 |
| Results of individual studies | 19 | For all outcomes, present, for each study: (a) summary statistics for each group (where appropriate) and (b) an effect estimate and its precision (e.g. confidence/credible interval), ideally using structured tables or plots. | Appendix pg. 21-27 |
| Results of syntheses | 20a | For each synthesis, briefly summarise the characteristics and risk of bias among contributing studies. | Pg. 10-12 |
|  | 20b | Present results of all statistical syntheses conducted. If meta-analysis was done, present for each the summary estimate and its precision (e.g., confidence/credible interval) and measures of statistical heterogeneity. If comparing groups, describe the direction of the effect. | Pg. 10-12 |
|  | 20c | Present results of all investigations of possible causes of heterogeneity among study results. | NA |
|  | 20d | Present results of all sensitivity analyses conducted to assess the robustness of the synthesized results. | NA |
| Reporting biases | 21 | Present assessments of risk of bias due to missing results (arising from reporting biases) for each synthesis assessed. | NA |
| Certainty of evidence | 22 | Present assessments of certainty (or confidence) in the body of evidence for each outcome assessed. | Pg. 10-12 |
| **DISCUSSION** | | |  |
| Discussion | 23a | Provide a general interpretation of the results in the context of other evidence. | Pg. 13-15 |
|  | 23b | Discuss any limitations of the evidence included in the review. | Pg. 16 |
|  | 23c | Discuss any limitations of the review processes used. | Pg. 16 |
|  | 23d | Discuss implications of the results for practice, policy, and future research. | Pg. 16 |
| **OTHER INFORMATION** | | |  |
| Registration and protocol | 24a | Provide registration information for the review, including register name and registration number, or state that the review was not registered. | Pg. 6 |
|  | 24b | Indicate where the review protocol can be accessed, or state that a protocol was not prepared. | Pg. 6 |
|  | 24c | Describe and explain any amendments to information provided at registration or in the protocol. | NA |
| Support | 25 | Describe sources of financial or non-financial support for the review, and the role of the funders or sponsors in the review. | Pg. 3, 18 |
| Competing interests | 26 | Declare any competing interests of review authors. | Pg. 18 |
| Availability of data, code, and other materials | 27 | Report which of the following are publicly available and where they can be found: template data collection forms; data extracted from included studies; data used for all analyses; analytic code; any other materials used in the review. | NA |

**Supplementary table 2.** Guidelines for Accurate and Transparent Health Estimates Reporting (GATHER) checklist

| **Item #** | **Checklist item** | **Reported on page #** |
| --- | --- | --- |
| **Objectives and funding** | | |
| **1** | Define the indicator(s), populations (including age, sex, and geographic entities), and time period(s) for which estimates were made. | Pg. 6-9 |
| **2** | List the funding sources for the work. | Pg. 2, 18 |
| **Data inputs** | | |
| *For all data inputs from multiple sources that are synthesized as part of the study:* | | |
| **3** | Describe how the data were identified and how the data were accessed. | Pg. 6-7 |
| **4** | Specify the inclusion and exclusion criteria. Identify all ad‐hoc exclusions. | Pg. 7-8 |
| **5** | Provide information on all included data sources and their main characteristics. For each data source used, report reference information or contact name/institution, population represented, data collection method, year(s) of data collection, sex and age range, diagnostic criteria or measurement method, and sample size, as relevant. | Pg. 10  Appendix pg. 21-27 |
| **6** | Identify and describe any categories of input data that have potentially important biases (e.g., based on characteristics listed in item 5). | NA |
| *For data inputs that contribute to the analysis but were not synthesized as part of the study:* | | |
| **7** | Describe and give sources for any other data inputs. | NA |
| *For all data inputs:* | | |
| **8** | Provide all data inputs in a file format from which data can be efficiently extracted (e.g., a spreadsheet rather than a PDF), including all relevant meta‐data listed in item 5. For any data inputs that cannot be shared because of ethical or legal reasons, such as third‐party ownership, provide a contact name or the name of the institution that retains the right to the data. | NA |
| **Data analysis** | | |
| **9** | Provide a conceptual overview of the data analysis method. A diagram may be helpful. | Pg. 8-9 |
| **10** | Provide a detailed description of all steps of the analysis, including mathematical formulae. This description should cover, as relevant, data cleaning, data pre‐processing, data adjustments and weighting of data sources, and mathematical or statistical model(s). | Pg. 8-9 |
| **11** | Describe how candidate models were evaluated and how the final model(s) were selected. | NA |
| **12** | Provide the results of an evaluation of model performance, if done, as well as the results of any relevant sensitivity analysis. | NA |
| **13** | Describe methods for calculating uncertainty of the estimates. State which sources of uncertainty were, and were not, accounted for in the uncertainty analysis. | NA |
| **14** | State how analytic or statistical source code used to generate estimates can be accessed. | NA |
| **Results and Discussion** | | |
| **15** | Provide published estimates in a file format from which data can be efficiently extracted. | Pg. 10-12 |
| **16** | Report a quantitative measure of the uncertainty of the estimates (e.g., uncertainty intervals) | Pg. 10-12 |
| **17** | Interpret results in light of existing evidence. If updating a previous set of estimates, describe the reasons for changes in estimates | Pg. 13-15 |
| **18** | Discuss limitations of the estimates. Include a discussion of any modelling assumptions or data limitations that affect interpretation of the estimates. | Pg. 16 |

**Supplementary table 3.** Search strategies used in each bibliographic database

| **Database** | **Search strategy** |
| --- | --- |
| EMBASE | #1 ((inject drug*) OR (injecting drug*) OR (injection drug*) OR (injecting substance*) OR (intravenous drug*) OR (intravenous substance*) OR (drug inject*) OR (drug use*) OR (drug abuse*) OR (people who inject*) OR (persons who inject*) OR PWID OR IDU OR IVDU OR (drug depend*) OR (substance use*) OR (substance misuse*) OR (substance abuse*) OR (drug addict*) OR (opioid substitution*) OR (opioid agonist*) OR (opioid maintenance*) OR (opiate substitution*) OR (opiate maintenance*) OR (opiate agonist*) OR OST OR OAT OR (methadone therap*) OR (methadone treat*) OR (methadone maintenance) OR (buprenorphine therap*) OR (buprenorphine treat*) OR (buprenorphine maintenance)).ti,ab,kw. **OR Injection drug user.sh OR Intravenous drug abuse.sh OR Opiate substitution treatment.sh**  #2 (abscess* OR cellulitis OR myositis OR (necrotising fasciitis) OR (necrotizing fasciitis) OR (skin and soft tissue infection*) OR SSTI* OR (injecting site infection*) OR (injection site infection*) OR (skin infection*) OR (cutaneous infection*) OR (soft tissue infection*) OR (soft tissue injur*) OR gangrene OR (leg ulcer*)).ti,ab,kw. **OR Abscess.sh OR Cellulitis.sh OR Skin infection.sh OR Soft tissue infection.sh**  #3  (endocarditis OR (septic thrombophlebitis) OR (mycotic aneurysm) OR (septic embolism) OR (epidural abscess*) OR meningitis OR (bone and joint infection*) OR (bone infection*) OR (joint infection*) OR osteomyelitis OR (septic arthritis)).ti,ab,kw. **OR Endocarditis.sh OR Central nervous system infection.sh OR Osteomyelitis.sh**  #4  ((bloodstream infection*) OR septicemia OR septicaemia OR sepsis OR (septic shock) OR bacteraemia OR bacteremia OR fungaemia OR fungemia OR candidaemia OR candidemia).ti,ab,kw. **OR Bacteremia.sh OR Fungemia.sh OR Candidiasis.sh**  #5  ((injecting related injury and disease) OR (injecting related infection*) OR (injection related infection*) OR (bacterial infection*) OR (fungal infection*) OR (severe infection*) OR (invasive infection*) OR (systemic infection*) OR (disseminated infection*) OR (critical illness*)).ti,ab,kw.  # #1 AND (#2 OR #3 OR #4 OR #5) |
| MEDLINE (PubMed) | #1 inject drug*[tiab] OR injecting drug*[tiab] OR injection drug*[tiab] OR injecting substance*[tiab] OR intravenous drug*[tiab] OR intravenous substance*[tiab] OR drug inject*[tiab] OR drug use*[tiab] OR drug abuse*[tiab] OR people who inject*[tiab] OR persons who inject*[tiab] OR PWID[tiab] OR IDU[tiab] OR IVDU[tiab] OR drug depend*[tiab] OR substance use*[tiab] OR substance misuse*[tiab] OR substance abuse*[tiab] OR drug addict*[tiab] OR opioid substitution*[tiab] OR opioid agonist*[tiab] OR opioid maintenance*[tiab] OR opiate substitution*[tiab] OR opiate maintenance*[tiab] OR opiate agonist*[tiab] OR OST[tiab] OR OAT[tiab] OR methadone therap*[tiab] OR methadone treat*[tiab] OR methadone maintenance[tiab] OR buprenorphine therap*[tiab] OR buprenorphine treat*[tiab] OR buprenorphine maintenance[tiab] OR **Substance Abuse, Intravenous[mh] OR Opiate Substitution Treatment[mh]**  #2 abscess*[tiab] OR **Abscess[mh]** OR cellulitis[tiab] OR **Cellulitis[mh]** OR myositis[tiab] OR necrotising fasciitis[tiab] OR necrotizing fasciitis[tiab] OR skin and soft tissue infection*[tiab] OR SSTI*[tiab] OR injecting site infection*[tiab] OR injection site infection*[tiab] OR skin infection*[tiab] OR cutaneous infection*[tiab] OR soft tissue infection*[tiab] OR soft tissue injur*[tiab] OR gangrene[tiab] OR leg ulcer*[tiab]  #3  endocarditis[tiab] OR **Endocarditis[mh]** OR septic thrombophlebitis[tiab] OR mycotic aneurysm[tiab] OR septic embolism[tiab] OR epidural abscess*[tiab] OR meningitis[tiab] OR **Central nervous system infection[mh]** OR bone and joint infection*[tiab] OR **Bone disease, infectious[mh]** OR bone infection*[tiab] OR joint infection*[tiab] OR osteomyelitis[tiab] OR **Osteomyelitis[mh]** OR septic arthritis[tiab]  #4  bloodstream infection*[tiab] OR septicemia[tiab] OR septicaemia[tiab] OR sepsis[tiab] OR septic shock[tiab] OR bacteraemia[tiab] OR bacteremia[tiab] OR **Bacteremia[mh]** OR fungaemia[tiab] OR fungemia[tiab] OR **Fungemia[mh]** OR candidaemia[tiab] OR candidemia[tiab] OR **Candidiasis[mh]**  #5  injecting related injury and disease[tiab] OR injecting related infection*[tiab] OR injection related infection*[tiab] OR bacterial infection*[tiab] OR fungal infection*[tiab] OR severe infection*[tiab] OR invasive infection*[tiab] OR systemic infection*[tiab] OR disseminated infection*[tiab] OR critical illness*[tiab]  # #1 AND (#2 OR #3 OR #4 OR #5) |
| Web of Science | #1 TS=(“inject drug*” OR “injecting drug*” OR “injection drug*” OR “injecting substance*” OR “intravenous drug*” OR “intravenous substance*” OR “drug inject*” OR “drug use*” OR “drug abuse*” OR “people who inject*” OR “persons who inject*” OR PWID OR IDU OR IVDU OR “drug depend*” OR “substance use*” OR “substance misuse*” OR “substance abuse*” OR “drug addict*” OR “opioid substitution*” OR “opioid agonist*” OR “opioid maintenance*” OR “opiate substitution*” OR “opiate maintenance*” OR “opiate agonist*” OR OST OR OAT OR “methadone therap*” OR “methadone treat*” OR “methadone maintenance” OR “buprenorphine therap*” OR “buprenorphine treat*” OR “buprenorphine maintenance”)  #2 TS=(abscess* OR cellulitis OR myositis OR “necrotising fasciitis” OR “necrotizing fasciitis” OR “skin and soft tissue infection*” OR SSTI* OR “injecting site infection*” OR “injection site infection*” OR “skin infection*” OR “cutaneous infection*” OR “soft tissue infection*” OR “soft tissue injur*” OR gangrene OR “leg ulcer*”)  #3  TS=(endocarditis OR “septic thrombophlebitis” OR “mycotic aneurysm” OR “septic embolism” OR “epidural abscess*” OR meningitis OR “bone and joint infection*” OR “bone infection*” OR “joint infection*” OR osteomyelitis OR “septic arthritis”)  #4  TS=(“bloodstream infection*” OR septicemia OR septicaemia OR sepsis OR “septic shock” OR bacteraemia OR bacteremia OR fungaemia OR fungemia OR candidaemia OR candidemia)  #5  TS=(“injecting related injury and disease” OR “injecting related infection*” OR “injection related infection*” OR “bacterial infection*” OR “fungal infection*” OR “severe infection*” OR “invasive infection*” OR “systemic infection*” OR “disseminated infection*” OR “critical illness*”)  # #1 AND (#2 OR #3 OR #4 OR #5) |
| PsycINFO | #1 TIAB(“inject drug*” OR “injecting drug*” OR “injection drug*” OR “injecting substance*” OR “intravenous drug*” OR “intravenous substance*” OR “drug inject*” OR “drug use*” OR “drug abuse*” OR “people who inject*” OR “persons who inject*” OR PWID OR IDU OR IVDU OR “drug depend*” OR “substance use*” OR “substance misuse*” OR “substance abuse*” OR “drug addict*” OR “opioid substitution*” OR “opioid agonist*” OR “opioid maintenance*” OR “opiate substitution*” OR “opiate maintenance*” OR “opiate agonist*” OR OST OR OAT OR “methadone therap*” OR “methadone treat*” OR “methadone maintenance” OR “buprenorphine therap*” OR “buprenorphine treat*” OR “buprenorphine maintenance”)  #2 TIAB(abscess* OR cellulitis OR myositis OR “necrotising fasciitis” OR “necrotizing fasciitis” OR “skin and soft tissue infection*” OR SSTI* OR “injecting site infection*” OR “injection site infection*” OR “skin infection*” OR “cutaneous infection*” OR “soft tissue infection*” OR “soft tissue injur*” OR gangrene OR “leg ulcer*”)  #3  TIAB(endocarditis OR “septic thrombophlebitis” OR “mycotic aneurysm” OR “septic embolism” OR “epidural abscess*” OR meningitis OR “bone and joint infection*” OR “bone infection*” OR “joint infection*” OR osteomyelitis OR “septic arthritis”)  #4  TIAB(“bloodstream infection*” OR septicemia OR septicaemia OR sepsis OR “septic shock” OR bacteraemia OR bacteremia OR fungaemia OR fungemia OR candidaemia OR candidemia)  #5  TIAB(“injecting related injury and disease” OR “injecting related infection*” OR “injection related infection*” OR “bacterial infection*” OR “fungal infection*” OR “severe infection*” OR “invasive infection*” OR “systemic infection*” OR “disseminated infection*” OR “critical illness*”)  # #1 AND (#2 OR #3 OR #4 OR #5) |

**Supplementary table 4.** List of extracted data items

| **STUDY-LEVEL DATA & SAMPLE CHARACTERISTICS** | |  |
| --- | --- | --- |
| **author** | First author surname |  |
| **year** | Year of publication |  |
| **country** | Country of report |  |
| **literature_type** | Type of literature | Abstract; Journal article; Report; Other |
| **study_design** | Design of the study | Cross-sectional study; Prospective cohort study; Retrospective cohort study; Data linkage study; Randomised controlled trial; Qualitative study; Other (specify) |
| **community_hospital** | Indicate whether study site(s) were based in the community or hospital. | Community; Hospital |
| **singlemulti_site** | Indicate whether there was a single or multiple study sites | Single site; Multiple sites |
| **setting** | Type of setting (e.g., NSP, OAT clinic, hospital, prison) | NSP; OAT clinic; Supervised injecting facility; Hospital inpatient; Hospital outpatient; Emergency department; Prison; Multiple (specify);Other (specify) |
| **setting_def** | Description of the study setting provided |  |
| **population** | Study population | People who inject drugs; People receiving OAT; People with drug-related hospitalisation; Other (specify) |
| **inclusion_exclusion** | Study inclusion/exclusion criteria |  |
| **inject_recency  (PWID sample)** | If study population is PWID, indicate how recently participants had injected | Past 12 months; Past 6 months; Past 3 months; Past month; Current; Other (specify) |
| **oat_recency  (OAT sample)** | If study population is people on OAT, indicate how recently participants had received OAT | Past 12 months; Past 6 months; Past 3 months; Past month; Current; Other (specify) |
| **subgroup_yn** | Indicate whether the study population is a subgroup/subpopulation of PWID or people on OAT | Yes; No |
| **subgroup_specify** | Specify |  |
| **iduoat_assessment** | Indicate how injecting or OAT status of participants was assessed | Self-report; ICD codes; Clinical record/assessment; Other (specify) |
| **n_overall** | Overall number of participants in which the outcome was assessed |  |
| **n_male** | Number of males in the overall sample |  |
| **%_male** | Percentage of males in the overall sample |  |
| **age** | Age (mean or median) of the overall sample |  |
| **age_mean_median** | Indicate whether value entered above is mean or median |  |
| **n_hcv** | Number of people in the overall sample with HCV infection |  |
| **%_hcv** | Percentage of the overall sample with HCV infection |  |
| **n_hiv** | Number of people in the overall sample with HIV infection |  |
| **%_hiv** | Percentage of the overall sample with HIV infection |  |
| **n_unstablehousing** | Number of people in the overall sample with unstable housing |  |
| **%_unstablehousing** | Percentage of people in the overall sample with unstable housing |  |
| **unstablehousing_definition** | Type of unstable housing reported | Homeless; Lack of rented/owned accommodation; Other |
| **unstablehousing_timeframe** | Timeframe of report for housing data | Lifetime; Past 12 months; Past 6 months; Past 3 months; Past month; Current; Other |
| **n_incarceration** | Number of people in the overall sample with incarceration history |  |
| **%_incarceration** | Percentage of people in the overall sample with incarceration history |  |
| **incarceration_timeframe** | Timeframe of report for incarceration data | Lifetime; Past 12 months; Past 6 months; Past 3 months; Past month; Current; Other |
| **n_unsterile** | Number of people in the overall sample who have used an unsterile needle or syringe | (Includes receptive sharing and re-use of one's own needle or syringe) |
| **%_unsterile** | Percentage of people in the overall sample who have used an unsterile needle or syringe |  |
| **unsterile_definition** | Type of data reported |  |
| **unsterile_timeframe** | Timeframe of report for unsterile data | Lifetime; Past 12 months; Past 6 months; Past 3 months; Past month; Other |
| **n_maindrug** | Number of people in the overall sample who report injecting X as their "main" drug |  |
| **%_maindrug** | Percentage of people in the overall sample who report injecting X as their "main" drug |  |
| **maindrug_specify** | Specify the type of drug reported | (e.g., heroin, amphetamines) |
| **n_injectfreq** | Number of people in the overall sample injecting at X frequency |  |
| **%_injectfreq** | Percentage of people in the overall sample injecting at X frequency |  |
| **injectfreq_definition** | Definition of injecting frequency | Daily or more; Weekly; Monthly |
| **n_oat** | Number of people in the overall sample receiving opioid agonist treatment (OAT) |  |
| **%_oat** | Percentage of people in the overall sample receiving OAT |  |
| **oat_timeframe** | Timeframe of report for OAT data | Lifetime; Past 12 months; Past 6 months; Past 3 months; Past month; Current; Other |
| **n_nsp** | Number of people in the overall sample visiting/accessing needle syringe programme (NSP) services |  |
| **%_nsp** | Percentage of people in the overall sample visiting/accessing NSP services |  |
| **nsp_timeframe** | Timeframe of report for NSP data | Lifetime; Past 12 months; Past 6 months; Past 3 months; Past month; Current; Other |
| **n_sif** | Number of people in the overall sample visiting/accessing a supervised injecting facility (SIF). | NB: may also be described as a drug consumption room (DCR) |
| **%_sif** | Percentage of people in the overall sample visiting/accessing a SIF |  |
| **sif_timeframe** | Timeframe of report for SIF data | Lifetime; Past 12 months; Past 6 months; Past 3 months; Past month; Current; Other |
| **Does the study report specific injecting risk behaviours?** | Other injecting risk behaviours  (that do not fall under the category of "use of unsterile needle/syringe"). | May include sharing/re-use of other injecting equipment (e.g., filter, spoon), licking needles prior to injecting, injecting in public, using non-sterile water source for drug dilution, cleaning of injection site and equipment prior to injecting |
| **OUTCOME (PREVALENCE)** |  |  |
| **study_id** |  |  |
| **outcome_number** | Outcome number |  |
| **outcome** | Type of outcome reported | Occurrence/experience; Hospitalisation; ED presentation; Other (specify) |
| **outcome_timeframe** | Timeframe of report of the outcome | Lifetime; Past 12 months; Past 6 months; Past 3 months; Past month; Current; Other (specify) |
| **infection_type** | Type of infection reported | Abscess; Cellulitis; Skin and soft tissue infection; Endocarditis; Bloodstream infection; Osteomyelitis; Septic arthritis; Sepsis; Other (specify); Multiple (specify) |
| **multiple_infectiontype_specify** | Specify if the outcome is a composite of multiple infection types |  |
| **infection_definition** | Infection definition |  |
| **infection_assessment** | Indicate how infection status was assessed (e.g., self-report, clinical records) | Self-report; ICD codes; Clinical record/assessment; Other (specify) |
| **n_overall** | Overall number of participants in which the outcome was assessed |  |
| **n_outcome** | Number of people with the outcome (infection) |  |
| **%_outcome** | Percentage of people with the outcome (infection) |  |
|  |  |  |
| **OUTCOME (INCIDENCE)** |  |  |
| **study_id** |  |  |
| **outcome_number** | Outcome number |  |
| **outcome** | Type of outcome reported | Occurrence/experience; Hospitalisation; ED presentation; Other (specify) |
| **infection_type** | Type of infection reported | Abscess; Cellulitis; Skin and soft tissue infection; Endocarditis; Bloodstream infection; Osteomyelitis; Septic arthritis; Sepsis; Other (specify); Multiple (specify) |
| **multiple_infectiontype_specify** | Specify if the outcome is a composite of multiple infection types |  |
| **infection_definition** | Infection definition |  |
| **infection_assessment** | Indicate how infection status was assessed | Self-report; ICD codes; Clinical record/assessment; Other (specify) |
| **n_overall** | Overall number of participants in which the outcome was assessed |  |
| **fu_month_total** | Total number of months of follow-up |  |
| **fu_mean_median** | Mean or median months of follow-up among participants |  |
| **mean_median** | Indicate whether value entered above is mean or median |  |
| **py_total** | Total person-years of follow-up |  |
| **n_outcome** | Number of participants with the outcome across the follow-up period |  |
| **rate_outcome  (per 1,000 PY FU)** | Rate of the outcome per 1,000 person-years of follow-up |  |
| **rate_outcome (per 10,000 PY FU)** | Rate of the outcome per 10,000 person-years of follow-up |  |

**Supplementary table 5.** Modified Joanna Briggs Institute (JBI) Critical Appraisal Checklist for prevalence studies

| **Joanna Briggs Institute checklist item** | **Modified Joanna Briggs Institute checklist** | **Author notes** |
| --- | --- | --- |
| *Item 1: Was the sample frame appropriate to address the target population?* | **1.** Was the sample frame appropriate to address the target population? (Yes/No/Unclear) | Sampling frames that satisfied the following conditions were considered appropriate:   1. Study inclusion/exclusion criteria does NOT lead to overrepresentation and/or omission of a particular subgroup that would typically be present within the target population, PWID (e.g., people who are HIV or HCV positive) 2. Recruitment of study participants occurred across different types of settings (e.g., recruitment from harm reduction facilities [NSP, OAT etc.] AND via street-based outreach, to promote appropriate representation of PWID not accessing harm reduction services). 3. Recruitment site(s) covered multiple geographical locations (e.g., multiple cities or states) and were NOT restricted to a single, small geographical location (e.g., a single city). |
| *Item 2: Were study participants sampled in an appropriate way?* | **2.** Were study participants sampled in an appropriate way? (Yes/No/Unclear) | ‘Yes’ if participants were sampled using probability (e.g., random, systematic, stratified) or census-based sampling methods. ‘No’ if participants were sampled using non-probability sampling methods (e.g., convenience, consecutive, snowball, respondent-driven sampling). |
| *Item 3: Was the sample size adequate?* | **3.** Was the sample size adequate? (Yes/No/Unclear) | ‘Yes’ if a sample size calculation was carried out and the target sample size was attained. ‘Unclear’ if no evidence of a sample size calculation. Large samples (e.g., large national survey) were assumed adequate. |
| *Item 4: Were the study subjects and setting described in detail?* | **4a.** Were the study subjects described in detail? (Yes/No/Unclear) | ‘Yes’ if data on both age and sex of study subjects was reported. ‘No’ if only age OR sex reported, or neither reported. |
| *Item 4: Were the study subjects and setting described in detail?* | **4b.** Was the study setting described in detail? (Yes/No/Unclear) | Description of the study setting was considered sufficiently detailed if the type, number, and location of recruitment site(s) was provided. |
| *Item 5: Was the data analysis conducted with sufficient coverage of the identified sample?* | **5.** Was the data analysis conducted with sufficient coverage of the identified sample? (Yes/No/Unclear) | Coverage of the identified sample was considered adequate if the following conditions were met:   1. Evidence that the response rate (proportion of eligible individuals approached to participate who were enrolled) did NOT differ significantly across subgroups of the target population, PWID 2. Evidence that the amount of missing data (proportion of enrolled study participants for whom data on the outcome was not obtained) did NOT differ significantly across subgroups of the target population, PWID |
| *Item 9: Was the response rate adequate, and if not, was the low response rate managed appropriately?* | **6.** Was the response rate adequate, and if not, was the low response rate managed appropriately? (Yes/No/Unclear) | ‘No’ if the authors reported a low response rate and/or large amount of missing data. |
| *N/A* | **Overall selection bias**  **(Yes/No/Unclear)** | An overall assessment of selection bias was derived using responses to Q1-Q6 of the modified checklist. We considered studies to be at risk of selection bias if one or more responses of ‘No’ were given for Q1-Q6. |
| *Item 6: Were valid methods used for the identification of the condition?* | **7.** Were objective methods used for identification of the condition? (Yes/No/Unclear) | ‘Yes’ if the outcome was measured using clinical records, clinical assessment and/or ICD codes. ‘No’ if the outcome was self-reported by study participants. |
| *Item 7: Was the condition measured in a standard, reliable way for all participants?* | **8.** Was the condition measured in a standard, reliable way for all participants? (Yes/No/Unclear) | ‘Yes’ if the outcome was measured using the same methods for all study participants. |
| *Item 8: Was there appropriate statistical analysis?* | **9.** Was there an appropriate statistical analysis? (Yes/No/Unclear) | ‘Yes’ if the authors reported a numerator (number of individuals with the outcome) and denominator (number of individuals assessed for the outcome), or proportion with the outcome. |
| *N/A* | **Overall misclassification bias (Yes/No/Unclear)** | An overall assessment of misclassification bias was derived using responses to Q7-Q9 of the modified checklist. We considered studies to be at risk of misclassification bias if one or more responses of ‘No’ were given for Q7-Q9. |

**Supplementary table 6.** Quality assessment of studies evaluating infection prevalence using a modified version of the Joanna Briggs Institute (JBI) Critical Appraisal Checklist for prevalence studies

| **Study** | **Selection bias** | | | | | | | | **Misclassification bias** | | | |
| --- | --- | --- | --- | --- | --- | --- | --- | --- | --- | --- | --- | --- |
|  | (1) Was the sample frame appropriate to address the target population? | (2) Were study participants sampled in an appropriate way? | (3) Was the sample size adequate? | (4a) Were the study subjects described in detail? | (4b) Was the study setting described in detail? | (5) Was data analysis conducted with sufficient coverage of the sample? | (6) Was the response rate and amount of missing data acceptable? | Overall assessm-ent of selection bias (Y/N) | (7) Were objective methods used for identification of the condition? | (8) Was the condition measured in a standard, reliable way for all participants? | (9) Was there an appropriate statistical analysis? | Overall assessm-ent of misclassi-fication bias (Y/N) |
| **Alexander, 2022^1^** | No | No | Unclear | No | Yes | Unclear | Unclear | Yes | No | Yes | Yes | Yes |
| **Ambekar, 2015^2^** | No | Yes | Unclear | Yes | Yes | Unclear | Yes | Yes | No | Yes | Yes | Yes |
| **Asher, 2019^3^** | No | No | Unclear | No | Yes | Unclear | Unclear | Yes | No | Yes | Yes | Yes |
| **Aslam, 2022^4^** | No | No | Unclear | No | Yes | Unclear | Unclear | Yes | Yes | Yes | Yes | No |
| **Axelsson, 2014^5^** | No | No | Unclear | Yes | Yes | Unclear | Unclear | Yes | No | Yes | Yes | Yes |
| **Baltes, 2020^6^** | No | No | Unclear | No | Yes | Unclear | Unclear | Yes | No | Yes | Yes | Yes |
| **Barocas, 2013^7^** | No | Unclear | Unclear | Yes | Yes | Unclear | Unclear | Yes | No | Yes | Yes | Yes |
| **Batisse, 2022^8^** | No | Yes | Unclear | No | Yes | Yes | Yes | Yes | Yes | Yes | Yes | No |
| **Benrubi, 2023^9^** | Yes | No | Unclear | Yes | Yes | Unclear | Unclear | Yes | No | Yes | Yes | Yes |
| **Bicket, 2020^10^** | No | No | Unclear | No | Yes | Unclear | Unclear | Yes | No | Yes | Yes | Yes |
| **Bonar, 2011^11^** | No | No | Unclear | Yes | Yes | Unclear | Unclear | Yes | No | Yes | Yes | Yes |
| **Bull-Otterson, 2020**^12^ | No | Yes | Yes | No | Yes | Yes | Yes | Yes | Yes | Yes | Yes | No |
| **Calderon-Villarreal, 2024^13^** | No | No | Unclear | Yes | Yes | Unclear | Unclear | Yes | No | Yes | Yes | Yes |
| **Colledge-Frisby, 2022^14^** | No | Yes | Yes | Yes | Yes | Yes | Yes | Yes | Yes | Yes | Yes | No |
| **Coull, 2021**^15^ | No | No | Unclear | No | Yes | Unclear | Unclear | Yes | No | Yes | Yes | Yes |
| **Curtis, 2023^16^** | No | Yes | Unclear | Yes | Yes | Unclear | Unclear | Yes | Yes | Yes | Yes | No |
| **Dahlman, 2015^17^** | No | No | Unclear | Yes | Yes | Unclear | Unclear | Yes | No | Yes | Yes | Yes |
| **Dahlman, 2017^18^** | No | Unclear | Unclear | Yes | Yes | Unclear | Unclear | Yes | No | Yes | Yes | Yes |
| **Dion, 2020^19^** | No | No | Unclear | Yes | Yes | Unclear | Unclear | Yes | No | Yes | Yes | Yes |
| **Frank, 2024^20^** | No | No | Unclear | Yes | Yes | Unclear | Unclear | Yes | No | Yes | Yes | Yes |
| **Frost, 2018**^21^ | No | No | Unclear | Yes | Yes | Unclear | Unclear | Yes | No | Yes | Yes | Yes |
| **Glick, 2021^22^** | No | No | Unclear | No | Yes | Unclear | No | Yes | No | Yes | Yes | Yes |
| **Goncalves, 2023^23^** | No | No | Unclear | Yes | Yes | Unclear | Unclear | Yes | No | Yes | Yes | Yes |
| **Heimer, 2015^24^** | No | No | Yes | Yes | Yes | Unclear | Unclear | Yes | No | Yes | Yes | Yes |
| **Horan, 2019^25^** | No | No | Unclear | Yes | Yes | Unclear | Unclear | Yes | No | Yes | Yes | Yes |
| **IBBS, 2018^26^** | No | No | Yes | No | Yes | Unclear | Unclear | Yes | No | Yes | Yes | Yes |
| **IDRS, 2010^27^** | Yes | No | Yes | Yes | Yes | Unclear | Unclear | Yes | No | Yes | Yes | Yes |
| **IDRS, 2011^28^** | Yes | No | Yes | Yes | Yes | Unclear | Unclear | Yes | No | Yes | Yes | Yes |
| **IDRS, 2012^29^** | Yes | No | Yes | Yes | Yes | Unclear | Unclear | Yes | No | Yes | Yes | Yes |
| **IDRS, 2013^30^** | Yes | No | Yes | Yes | Yes | Unclear | Unclear | Yes | No | Yes | Yes | Yes |
| **IDRS, 2014^31^** | Yes | No | Yes | Yes | Yes | Unclear | Unclear | Yes | No | Yes | Yes | Yes |
| **IDRS, 2015^32^** | Yes | No | Yes | Yes | Yes | Unclear | Unclear | Yes | No | Yes | Yes | Yes |
| **IDRS, 2016^33^** | Yes | No | Yes | Yes | Yes | Unclear | Unclear | Yes | No | Yes | Yes | Yes |
| **IDRS, 2017^34^** | Yes | No | Yes | Yes | Yes | Unclear | Unclear | Yes | No | Yes | Yes | Yes |
| **IDRS, 2018^35^** | Yes | No | Yes | Yes | Yes | Unclear | Unclear | Yes | No | Yes | Yes | Yes |
| **IDRS, 2019^36^** | Yes | No | Yes | Yes | Yes | Unclear | Unclear | Yes | No | Yes | Yes | Yes |
| **IDRS, 2020^37^** | Yes | No | Yes | Yes | Yes | Unclear | Unclear | Yes | No | Yes | Yes | Yes |
| **IDRS, 2021^38^** | Yes | No | Yes | Yes | Yes | Unclear | Unclear | Yes | No | Yes | Yes | Yes |
| **IDRS, 2022^39^** | Yes | No | Yes | Yes | Yes | Unclear | Unclear | Yes | No | Yes | Yes | Yes |
| **IDRS, 2023^40^** | Yes | No | Yes | Yes | Yes | Unclear | Unclear | Yes | No | Yes | Yes | Yes |
| **IDRS, 2024^41^** | Yes | No | Yes | Yes | Yes | Unclear | Unclear | Yes | No | Yes | Yes | Yes |
| **Ivan, 2016^42^** | No | No | Unclear | No | Yes | Unclear | Unclear | Yes | No | Yes | Yes | Yes |
| **Johnson, 2013^43^** | No | Unclear | Unclear | No | Yes | Unclear | Unclear | Yes | No | Yes | Yes | Yes |
| **Jorgensen, 2024^44^** | No | No | Unclear | Yes | Yes | Unclear | Unclear | Yes | No | Yes | Yes | Yes |
| **Lee, 2013^45^** | No | No | Unclear | Yes | Yes | Unclear | Unclear | Yes | No | Yes | Yes | Yes |
| **Li, 2021^46^** | No | Yes | Unclear | No | Yes | Yes | Yes | Yes | Yes | Yes | Yes | No |
| **Maloney, 2010^47^** | No | No | Unclear | No | No | Unclear | Unclear | Yes | No | Unclear | Yes | Yes |
| **Marks, 2024^48^** | No | No | Unclear | Yes | Yes | Unclear | Unclear | Yes | No | Yes | Yes | Yes |
| **McMahan, 2020^49^** | No | No | Unclear | Yes | Yes | Unclear | Unclear | Yes | No | Yes | Yes | Yes |
| **Megerian, 2024^50^** | No | No | Unclear | Yes | Yes | Unclear | Unclear | Yes | No | Yes | Yes | Yes |
| **Mezaache, 2021^51^** | No | No | Unclear | Yes | Yes | Unclear | Unclear | Yes | No | Yes | Yes | Yes |
| **Morin, 2020^52^** | No | Yes | Yes | No | Yes | Yes | Yes | Yes | Yes | Yes | Yes | No |
| **NESI, 2014^53^** | No | No | Yes | Yes | Yes | Yes | Yes | Yes | No | Yes | Yes | Yes |
| **NESI, 2016^53^** | No | No | Yes | Yes | Yes | Yes | Yes | Yes | No | Yes | Yes | Yes |
| **NESI, 2018^53^** | No | No | Yes | Yes | Yes | Yes | Yes | Yes | No | Yes | Yes | Yes |
| **NESI, 2020^53^** | No | No | Yes | Yes | Yes | Yes | Yes | Yes | No | Yes | Yes | Yes |
| **NESI, 2023^54^** | No | No | Yes | Yes | Yes | Yes | Yes | Yes | No | Yes | Yes | Yes |
| **Noroozi, 2018^55^** | No | No | Unclear | Yes | Yes | Yes | Yes | Yes | No | Yes | Yes | Yes |
| **Ojha, 2014^56^** | No | Yes | Unclear | Yes | Yes | Unclear | Unclear | Yes | No | Yes | Yes | Yes |
| **Ozga, 2021^57^** | No | No | Yes | Yes | Yes | Unclear | Unclear | Yes | No | Yes | Yes | Yes |
| **Panda, 2014^58^** | Yes | Yes | Yes | Yes | Yes | Unclear | Unclear | Unclear | No | Yes | Yes | Yes |
| **Perri, 2021^59^** | No | No | Unclear | No | Yes | Unclear | Unclear | Yes | No | Yes | Yes | Yes |
| **Peyriere, 2013^60^** | No | Yes | Unclear | No | Yes | Yes | Yes | Yes | Yes | Yes | Yes | No |
| **Roux, 2021^61^** | No | No | Unclear | Yes | Yes | Unclear | Unclear | Yes | No | Yes | Yes | Yes |
| **Roux, 2022^62^** | No | No | Unclear | Yes | Yes | Unclear | Unclear | Yes | No | Yes | Yes | Yes |
| **Schneider, 2022^63^** | No | No | Unclear | Yes | Yes | Unclear | Unclear | Yes | No | Yes | Yes | Yes |
| **Smith, 2015^64^** | No | No | Unclear | Yes | Yes | Unclear | Unclear | Yes | Yes | Yes | Yes | No |
| **Srivastava, 2023^65^** | No | No | Unclear | Yes | Yes | Yes | Yes | Yes | Yes | Yes | Yes | No |
| **Summers, 2018^66^** | No | No | Unclear | Yes | Yes | Unclear | Unclear | Yes | No | Yes | Yes | Yes |
| **Syvertsen, 2014^67^** | No | No | Unclear | Yes | Yes | Unclear | Unclear | Yes | No | Yes | Yes | Yes |
| **Taylor, 2019^68^** | No | No | Unclear | Yes | Yes | Unclear | Unclear | Yes | No | Yes | Yes | Yes |
| **UAM, 2017^69^** | Yes | No | Yes | No | Yes | Yes | Yes | Yes | No | Yes | Yes | Yes |
| **UAM, 2018^70^** | Yes | No | Yes | No | Yes | Yes | Yes | Yes | No | Yes | Yes | Yes |
| **UAM, 2019^71^** | Yes | No | Yes | No | Yes | Yes | Yes | Yes | No | Yes | Yes | Yes |
| **UAM, 2020^72^** | Yes | No | Yes | No | Yes | Yes | Yes | Yes | No | Yes | Yes | Yes |
| **UAM, 2021^73^** | Yes | No | Yes | No | Yes | Yes | Yes | Yes | No | Yes | Yes | Yes |
| **Wright, 2021^74^** | No | No | Unclear | No | Yes | Unclear | Unclear | Yes | No | Yes | Yes | Yes |
| **Wurcel, 2018^75^** | No | No | Unclear | Yes | Yes | Unclear | Unclear | Yes | No | Yes | Yes | Yes |
| **Yen, 2014^76^** | No | No | Unclear | Yes | Yes | Unclear | Unclear | Yes | No | Yes | Yes | Yes |
| **Zimmerman, 2016^77^** | No | No | Unclear | Yes | Yes | Unclear | Unclear | Yes | No | Yes | Yes | Yes |

Abbreviations: IDRS: Illicit Drug Reporting System; IBBS: Integrated Biological Behavioural Surveillance; NESI: Needle Exchange Surveillance Initiative; UAM: Unlinked Anonymous Monitoring Survey

**Supplementary table 7.** Modified Newcastle-Ottawa Scale (NOS) for cohort studies

| **Newcastle-Ottawa Scale item** | **Modified Newcastle-Ottawa Scale** | **Author notes** |
| --- | --- | --- |
| **Selection** | | |
| *Item 1: Representativeness of the exposed cohort*   1. *Truly representative* ★ 2. *Somewhat representative* ★ 3. *Selected group of users* 4. *No description of derivation of cohort* | **1**. Representativeness of the exposed cohort   1. Truly representative ★ 2. Somewhat representative ★ 3. Selected group of users 4. No description of derivation of cohort | The cohort was considered ‘Truly representative’ (one star awarded) if the following criteria were satisfied: (1) study inclusion/exclusion criteria does NOT lead to overrepresentation and/or omission of a PWID subgroup (e.g., people who are HIV positive); (2) recruitment of study participants occurred across different types of settings; and (3) recruitment site(s) covered multiple geographical locations (e.g., multiple cities or states) and were NOT restricted to a single, small geographical location (e.g., a single city).  The cohort was considered ‘Somewhat representative’ (one star awarded) if only one or two of the above criteria were met.  The cohort was considered a ‘Selected group’ (no stars awarded) if restricted to PWID of a certain sex, age, HIV status, main drug injected etc.  ‘No description’ (no stars awarded) if the cohort was not described or described in limited detail. |
| *Item 2: Selection of non-exposed cohort*   1. *Drawn from same community as exposed cohort* ★ 2. *Drawn from different source* 3. *No description of derivation of non-exposed cohort* | Omitted | Case-control studies were not included in the present review; hence this item was omitted. |
| *Item 3: Ascertainment of exposure*   1. *Secure record* ★ 2. *Structured interview* ★ 3. *Written self-report* 4. *No description* | **2.** Ascertainment of exposure (injecting drug use or OAT)   1. Medical record and/or assessment ★ 2. Self-report 3. No description | One star was awarded if injecting and/or OAT status of study subjects was ascertained via medical records and/or assessment by a trained clinician or researcher. No stars were awarded if injecting and/or OAT status was only self-reported by subjects, or if the method of determining participant injecting and/or OAT status was not reported or ambiguous. |
| *Item 4: Demonstration that the outcome of interest was not present at start of study*   1. *Yes* ★ 2. *No* | **3.** Demonstration that the outcome (infection) of interest was not present at start of study   1. Yes ★ 2. No | One star was awarded if it was stated or demonstrated that study subjects did not have the infection of interest (e.g., abscess) at the start of the study (including exclusion of individuals with current infection as part of the study inclusion/exclusion criteria). |
| **Comparability** | | |
| *Item 1: Comparability of cohorts on the basis of the design or analysis*   1. *Study controls for most important factor* ★ 2. *Study controls for any additional factor* ★ | Omitted | Case-control studies were not included in the present review; hence this item was omitted. |
| **Outcome** | | |
| *Item 1: Assessment of outcome*   1. *Independent blind assessment* ★ 2. *Record linkage* ★ 3. *Self-report* 4. *No description* | **1.** Assessment of outcome (infection)   1. Independent blind assessment ★ 2. Record linkage ★ 3. Self-report 4. No description | One star was awarded if the presence of infection was ascertained via medical records and/or assessment by a trained clinician or researcher. No stars were awarded if infection was only self-reported by subjects, or if the method of determining infection status was not reported or ambiguous. |
| *Item 2: Was follow-up long enough for outcomes to occur?*   1. *Yes* ★ 2. *No* | **2.** Was follow-up long enough for outcomes (infection) to occur?   1. Yes ★ 2. No | One star was awarded if the median length of follow-up among study subjects was at least __months. |
| *Item 3: Adequacy of follow-up of cohorts*   1. *Complete follow up (all subjects accounted for)* ★ 2. *Subjects lost to follow up unlikely to introduce bias (small number lost to follow up)* ★ 3. *Follow up rate less than __%* 4. *No statement* | **3.** Adequacy of follow-up of cohort   1. Complete follow up (all subjects accounted for) ★ 2. Subjects lost to follow up unlikely to introduce bias (small number lost to follow up) ★ 3. Follow up rate less than __% 4. No statement | One star was awarded if no study subjects were lost to follow up, or if the proportion of subjects lost to follow up was small and unlikely to introduce bias. No stars were awarded if the authors reported a follow-up rate of less than __%, or if no information was provided regarding rate of follow up. |
| **Overall rating** | | |
| Overall star rating of 0-9 stars | Overall star rating of 0-6 stars | An overall star rating was derived by summing the number of stars awarded across all items of the modified scale, with a maximum score of 6 stars. |

**Supplementary table 8.** Quality assessment of studies evaluating infection incidence using a modified version of the Newcastle-Ottawa Scale (NOS) for cohort studies

| **Study** | **Selection** | | | **Outcome** | | | **Overall rating (out of 6)** |
| --- | --- | --- | --- | --- | --- | --- | --- |
|  | 1. Representativeness of cohort | 2. Ascertainment of exposure (injecting or OAT) | 3. Demonstration that the outcome (infection) was not present at start of study | 1. Assessment of outcome (infection) | 2. Length of follow-up | 3. Adequacy of follow-up |  |
| **Bonacci, 2022^78^** | ★ | ★ | ★ | ★ | ★ | ★ | ★★★★★★ |
| **Calderon-Villarreal, 2024^13^** | ★ | ★ | ★ | ★ | ★ | ★ | ★★★★★★ |
| **Colledge-Frisby, 2022^14^** | ★ | ★ | ★ | ★ | ★ | ★ | ★★★★★★ |
| **Curtis, 2023^16^** | ★ | ★ | ★ | ★ | ★ | ★ | ★★★★★★ |
| **Dahlman, 2018^79^** | ★ | ★ | ★ | ★ | ★ | ★ | ★★★★★★ |
| **Figgatt, 2023^80^** | ★ | ★ | ★ | ★ | ★ | ★ | ★★★★★★ |
| **Nambiar, 2017^81^** | ★ | ★ | ★ | ★ | ★ | ★ | ★★★★★★ |
| **Vanichseni, 2015^82^** | ★ | ★ | ★ | ★ | ★ | ★ | ★★★★★★ |
| **Wang, 2023^83^** | ★ | ★ | ★ | ★ | ★ | ★ | ★★★★★★ |

★= star not awarded;★= star awarded

**Supplementary table 9.** Characteristics of included studies assessing the prevalence of injecting-related infection (*K*= 78)

| **Study** | **Country** | **Study design** | **Population** | **Sample size** | **Sampling method(s)** | **Setting(s)** | **Outcome(s) and method of measurement** |
| --- | --- | --- | --- | --- | --- | --- | --- |
| Alexander, 2022^1^ | USA | Cross-sectional study | PWID | 77 | Convenience sampling | Drug treatment clinic (Medically managed drug withdrawal facility) | Endocarditis, skin and soft tissue infection  (Self-report) |
| Ambekar, 2015^2^ | India | Cross-sectional study | PWID  (Males only) | 902 | Random sampling | Needle syringe programs (NSPs) | Abscess  (Self-report) |
| Asher, 2019^3^ | USA | Retrospective cohort study | PWID | 541 | Respondent-driven sampling (RDS) and convenience sampling | Population/community-based | Abscess  (Self-report) |
| Aslam, 2022^4^ | India | Cross-sectional study | PWID | 217 | Convenience sampling | Drug treatment clinics | Skin and soft tissue infection  (Clinical record/assessment) |
| Axelsson, 2014^5^ | Denmark | Cross-sectional study | PWID | 206 | Convenience sampling | Supervised injecting facilities | Endocarditis  (Self-report) |
| Baltes, 2020^6^ | USA | Cross-sectional study | PWID | 80 | Respondent-driven sampling (RDS) | Population/community-based (rural) | Skin and soft tissue infection  (Self-report) |
| Barocas, 2013^7^ (abstract only) | USA | Cross-sectional study | PWID | 533 | Convenience sampling | Needle syringe programs (NSPs) | Endocarditis, skin and soft tissue infection  (Self-report) |
| Batisse, 2022^8^ | France | Retrospective cohort study | PWID | 186 | Census of all cases of chemsex | Population/community-based | Any injecting-related infection  (Clinical record/assessment) |
| Benrubi, 2023^9^ | USA | Cross-sectional study | PWID | 297 | Convenience sampling | Population/community-based | Skin and soft tissue infection  (Self-report) |
| Bicket, 2020^10^ | USA | Cross-sectional study | PWID | 203 | Convenience sampling | Needle syringe programs (NSPs) | Abscess, endocarditis, skin and soft tissue infection  (Self-report) |
| Bonar, 2011^11^ | USA | Cross-sectional study | PWID | 91 | Convenience sampling | Needle syringe programs (NSPs) | Any injecting-related infection  (Self-report) |
| Bull-Otterson, 2020^12^ | USA | Data linkage study | PWID | 844,242 | Census of health insurance database. | Population/community-based | Abscess, cellulitis, endocarditis, osteomyelitis  (ICD codes) |
| Calderon-Villarreal, 2024^13^ | USA, Mexico | Prospective cohort study | PWID | 647 | Convenience sampling | Population/community-based | Abscess  (Self-report) |
| Coull, 2021^15^ | Scotland | Cross-sectional study | PWID | 128 | Convenience sampling | Needle syringe programs (NSPs) and opioid agonist treatment (OAT) clinics | Abscess  (Self-report) |
| Dahlman, 2015^17^ | Sweden | Cross-sectional study | PWID | 80 | Convenience sampling | Needle syringe program (NSP) | Skin and soft tissue infection  (Self-report) |
| Dahlman, 2017^18^ | USA | Cross-sectional study | PWID | 201 | Convenience sampling | Population/community-based | Skin and soft tissue infection  (Self-report) |
| Dion, 2020^19^ | USA | Cross-sectional study | PWID | 141 | Convenience sampling | Needle syringe programs (NSPs) | Skin and soft tissue infection  (Self-report) |
| Frank, 2024^20^ | USA | Cross-sectional study | PWID | 822 | Convenience sampling | Needle syringe programs (NSPs) | Abscess, endocarditis, bloodstream infection  (Self-report) |
| Frost, 2018^21^ | USA | Cross-sectional study | People who inject opioids | 436 | Convenience sampling | Needle syringe programs (NSPs) | Abscess, skin and soft tissue infection  (Self-report) |
| Glick, 2021^22^ | USA | Cross-sectional study | People who inject opioids | 720 | Convenience sampling | Needle syringe programs (NSPs) | Bloodstream infection, endocarditis, skin and soft tissue infection  (Self-report) |
| Goncalves, 2023^23^ | France | Cross-sectional study | PWID | 140 | Convenience sampling | Harm reduction services | Abscess  (Self-report) |
| Heimer, 2015^24^ | Lebanon | Cross-sectional study | PWID | 390 | Respondent-driven sampling (RDS) | Population/community-based | Abscess  (Self-report) |
| Horan, 2019^25^ | Ireland | Cross-sectional study | PWID | 51 | Convenience sampling | Homeless services, needle syringe programs, other drug treatment services | Skin and soft tissue infection  (Self-report) |
| IBBS, 2018^26^ | Mauritius | Cross-sectional study | PWID | 500 | Respondent-driven sampling (RDS) | Population/community-based | Abscess  (Self-report) |
| IDRS, 2010^27^ | Australia | Cross-sectional study | PWID | 902 | Convenience sampling | Population/community-based | Any injecting-related infection  (Self-report) |
| IDRS, 2011^28^ | Australia | Cross-sectional study | PWID | 868 | Convenience sampling | Population/community-based | Any injecting-related infection  (Self-report) |
| IDRS, 2012^29^ | Australia | Cross-sectional study | PWID | 924 | Convenience sampling | Population/community-based | Abscess, cellulitis, endocarditis  (Self-report) |
| IDRS, 2013^30^ | Australia | Cross-sectional study | PWID | 887 | Convenience sampling | Population/community-based | Any injecting-related infection  (Self-report) |
| IDRS, 2014^31^ | Australia | Cross-sectional study | PWID | 898 | Convenience sampling | Population/community-based | Any injecting-related infection  (Self-report) |
| IDRS, 2015^32^ | Australia | Cross-sectional study | PWID | 888 | Convenience sampling | Population/community-based | Any injecting-related infection  (Self-report) |
| IDRS, 2016^33^ | Australia | Cross-sectional study | PWID | 877 | Convenience sampling | Population/community-based | Any injecting-related infection  (Self-report) |
| IDRS, 2017^34^ | Australia | Cross-sectional study | PWID | 888 | Convenience sampling | Population/community-based | Any injecting-related infection  (Self-report) |
| IDRS, 2018^35^ | Australia | Cross-sectional study | PWID | 905 | Convenience sampling | Population/community-based | Any injecting-related infection  (Self-report) |
| IDRS, 2019^36^ | Australia | Cross-sectional study | PWID | 902 | Convenience sampling | Population/community-based | Bloodstream infection, endocarditis, osteomyelitis, septic arthritis, skin and soft tissue infection  (Self-report) |
| IDRS, 2020^37^ | Australia | Cross-sectional study | PWID | 884 | Convenience sampling | Population/community-based | Bloodstream infection, osteomyelitis, septic arthritis, skin and soft tissue infection  (Self-report) |
| IDRS, 2021^38^ | Australia | Cross-sectional study | PWID | 888 | Convenience sampling | Population/community-based | Bloodstream infection, endocarditis, osteomyelitis, septic arthritis, skin and soft tissue infection  (Self-report) |
| IDRS, 2022^39^ | Australia | Cross-sectional study | PWID | 879 | Convenience sampling | Population/community-based | Bloodstream infection, endocarditis, osteomyelitis, septic arthritis, skin and soft tissue infection  (Self-report) |
| IDRS, 2023^40^ | Australia | Cross-sectional study | PWID | 817 | Convenience sampling | Population/community-based | Skin and soft tissue infection, any injecting-related infection  (Self-report) |
| IDRS, 2024^41^ | Australia | Cross-sectional study | PWID | 877 | Convenience sampling | Population/community-based | Skin and soft tissue infection, any injecting-related infection  (Self-report) |
| Ivan, 2016^42^ | Australia | Prospective cohort study | PWID | 45 | Convenience sampling | Integrated primary care and drug treatment service | Abscess, bloodstream infection, cellulitis  (Self-report) |
| Johnson, 2013^43^ (abstract only) | USA | Cross-sectional study | PWID | 81 | Convenience sampling | Needle syringe program (NSP) | Abscess  (Self-report) |
| Jorgensen, 2024^44^ | Sweden | Prospective cohort study | PWID | 61 | Convenience sampling | Needle syringe program (NSP) | Skin and soft tissue infection  (Self-report) |
| Lee, 2013^45^ | Thailand | Cross-sectional study | PWID | 430 | Convenience sampling | Population/community-based | Skin and soft tissue infection  (Self-report) |
| Li, 2021^46^ | USA | Retrospective cohort study | People who inject methamphetamine | 48 | Census of patients of a medical centre | Primary care practices, community health centres, drug treatment services | Abscess, bloodstream infection, osteomyelitis, septic arthritis  (Clinical record/assessment) |
| Maloney, 2010^47^  (abstract only) | Ireland | Cross-sectional study | PWID receiving OAT | 70 | Convenience sampling | Opioid agonist treatment (OAT) clinic | Abscess  (Self-report) |
| Marks, 2024^48^ | USA | Cross-sectional study | PWID | 728 | Convenience sampling | Drug treatment clinics | Abscess, cellulitis, endocarditis, bloodstream infection, any injecting-related infection  (Self-report) |
| McMahan, 2020^49^ | USA | Cross-sectional study | PWID | 583 | Convenience sampling | Needle syringe programs (NSPs) | Any injecting-related infection  (Self-report) |
| Megerian, 2024^50^ | USA | Cross-sectional study | PWID | 563 | Convenience sampling | Needle syringe programs (NSPs) | Skin and soft tissue infection, endocarditis  (Self-report) |
| Mezaache, 2021^51^ | France | Cross-sectional study | People who inject opioids | 557 | Convenience sampling | Harm reduction services, drug treatment clinics, online internet forums | Abscess, bloodstream infection  (Self-report) |
| Morin, 2020^52^ | Canada | Data linkage study | People receiving OAT | 55,924 | Census of all patients receiving opioid agonist treatment. | Population/community-based | Endocarditis, osteomyelitis, septic arthritis  (Clinical record/assessment) |
| NESI, 2014^53^ | Scotland | Cross-sectional study | PWID | 2,344 | Convenience sampling | Needle syringe programs (NSPs) | Skin and soft tissue infection  (Self-report) |
| NESI, 2016^53^ | Scotland | Cross-sectional study | PWID | 2,696 | Convenience sampling | Needle syringe programs (NSPs) | Skin and soft tissue infection  (Self-report) |
| NESI, 2018^53^ | Scotland | Cross-sectional study | PWID | 2,130 | Convenience sampling | Needle syringe programs (NSPs) | Skin and soft tissue infection  (Self-report) |
| NESI, 2020^53^ | Scotland | Cross-sectional study | PWID | 2,435 | Convenience sampling | Needle syringe programs (NSPs) | Skin and soft tissue infection  (Self-report) |
| NESI, 2023^54^ | Scotland | Cross-sectional study | PWID | 2,046 | Convenience sampling | Needle syringe programs (NSPs) | Skin and soft tissue infection  (Self-report) |
| Noroozi, 2018^55^ | Iran | Cross-sectional study | PWID | 500 | Convenience sampling | Harm reduction centres | Skin and soft tissue infection  (Self-report) |
| Ojha, 2014^56^ | Nepal | Cross-sectional study | PWID | 300 | Convenience sampling | Drug treatment services including rehabilitation centres, drug treatment clinics, opioid agonist treatment (OAT) clinics, counselling services | Skin and soft tissue infection  (Self-report) |
| Ozga, 2021^57^ | USA | Cross-sectional study | PWID | 494 | Respondent-driven sampling (RDS) | Population/community-based | Abscess  (Self-report) |
| Panda, 2014^58^ | India | Cross-sectional study | PWID | 1,155 | Random sampling | Non-government organisations providing harm reduction services | Skin and soft tissue infection  (Self-report) |
| Perri, 2021^59^ (abstract only) | USA | Randomised controlled trial | PWID | 56 | Convenience sampling | Needle syringe program (NSP) | Abscess, skin and soft tissue infection  (Self-report) |
| Peyriere, 2013^60^ | France | Prospective cohort study | PWID | 383 | Census of all cases of morphine sulfate abuse recorded in a drug monitoring system | Population/community-based | Any injecting-related infection  (Clinical record/assessment) |
| Roux, 2021^61^ | Bulgaria, Greece, Portugal, Romania | Cross-sectional study | PWID | 307 | Convenience sampling | Non-government organisations providing harm reduction services | Abscess  (Self-report) |
| Roux, 2022^62^ | France | Prospective cohort study | PWID | 662 | Convenience sampling | Drug consumption rooms, other harm reduction services | Abscess  (Self-report) |
| Schneider, 2022^63^ | USA | Non-randomised trial | PWID  (Female sex workers only) | 114 | Convenience sampling | Population/community-based | Skin and soft tissue infection  (Self-report) |
| Smith, 2015^64^ | USA | Cross-sectional study | PWID | 152 | Convenience sampling | Needle syringe program (NSP) | Abscess  (Clinical record/assessment) |
| Srivastava, 2023^65^  (abstract only) | USA | Retrospective cohort study | PWID (Veterans) | 216 | Census of patients of a medical centre | Primary care/general practice | Skin and soft tissue infection, bloodstream infection, osteomyelitis, septic arthritis  (ICD codes) |
| Summers, 2018^66^ | USA | Cross-sectional study | People who inject heroin | 145 | Convenience sampling | Needle syringe programs (NSPs) | Abscess  (Self-report) |
| Syvertsen, 2014^67^ | Kenya | Cross-sectional study | PWID | 150 | Snowball sampling | Population/community-based | Abscess  (Self-report) |
| Taylor, 2019^68^ | Portugal | Cross-sectional study | PWID | 72 | Convenience sampling | Population/community-based | Any injecting-related infection  (Self-report) |
| UAM, 2017^69^ | England, Northern Ireland, Wales | Cross-sectional study | PWID | 1,483 | Convenience sampling | Drug treatment services | Skin and soft tissue infection  (Self-report) |
| UAM, 2018^70^ | England, Northern Ireland, Wales | Cross-sectional study | PWID | 1,568 | Convenience sampling | Drug treatment services | Skin and soft tissue infection  (Self-report) |
| UAM, 2019^71^ | England, Northern Ireland, Wales | Cross-sectional study | PWID | 1,913 | Convenience sampling | Drug treatment services | Skin and soft tissue infection  (Self-report) |
| UAM, 2020^72^ | England, Northern Ireland, Wales | Cross-sectional study | PWID | 485 | Convenience sampling | Drug treatment services | Skin and soft tissue infection  (Self-report) |
| UAM, 2021^73^ | England, Northern Ireland, Wales | Cross-sectional study | PWID | 803 | Convenience sampling | Drug treatment services | Skin and soft tissue infection  (Self-report) |
| Wright, 2021^74^ | England | Cross-sectional study | PWID | 284 | Convenience sampling | Drug treatment services and homeless services | Skin and soft tissue infection  (Self-report) |
| Wurcel, 2018^75^ | USA | Cross-sectional study | PWID | 298 | Convenience sampling | Needle syringe programs (NSPs) and drug treatment services | Abscess  (Self-report) |
| Yen, 2014^76^ | Taiwan | Cross-sectional study | People receiving OAT | 827 | Convenience sampling | Opioid agonist treatment (OAT) clinics | Abscess  (Self-report) |
| Zimmerman, 2016^77^ | Germany | Cross-sectional study | PWID | 2,077 | Convenience sampling | Drug treatment services | Endocarditis  (Self-report) |

Abbreviations: PWID: people who inject drugs; OAT: opioid agonist treatment; IDRS: Illicit Drug Reporting System; NESI: Needle Exchange Surveillance Initiative; NSP, needle and syringe program; UAM: Unlinked Anonymous Monitoring Survey. *K*= number of studies.

**Supplementary table 10.** Studies evaluating the incidence of infection, hospitalisation for infection, emergency department presentation for infection, and mortality attributable to infection (*K*=9)

| **Study** | **Method of infection assessment** | **Outcome** | **Total person-years (PY) of follow-up** | **Sample size*** | **Number of males (%)** | **Mean or median age** | **Number with outcome** | **Incidence per 1,000 PY follow-up** |
| --- | --- | --- | --- | --- | --- | --- | --- | --- |
| Bonacci, 2022^78^ | Clinical record/assessment | Incidence of skin and soft tissue infection | 127 | 496 | NR | NR | 129 | 1,015.7 |
| Bonacci, 2022^78^ | Clinical record/assessment | Incidence of endocarditis | 127 | 496 | NR | NR | 22 | 173.2 |
| Bonacci, 2022^78^ | Clinical record/assessment | Incidence of bloodstream infection | 127 | 496 | NR | NR | 49 | 385.8 |
| Bonacci, 2022^78^ | Clinical record/assessment | Incidence of osteomyelitis | 127 | 496 | NR | NR | 20 | 157.5 |
| Bonacci, 2022^78^ | Clinical record/assessment | Incidence of hospitalisation for skin and soft tissue infection | 127 | 100 | NR | NR | 38 | 299.2 |
| Bonacci, 2022^78^ | Clinical record/assessment | Incidence of hospitalisation for endocarditis | 127 | 100 | NR | NR | 19 | 149.6 |
| Bonacci, 2022^78^ | Clinical record/assessment | Incidence of hospitalisation for bloodstream infection | 127 | 100 | NR | NR | 43 | 338.6 |
| Bonacci, 2022^78^ | Clinical record/assessment | Incidence of hospitalisation for osteomyelitis | 127 | 100 | NR | NR | 12 | 94.5 |
| Bonacci, 2022^78^ | Clinical record/assessment | Incidence of emergency department presentation for skin and soft tissue infection | 127 | 207 | NR | NR | 64 | 503.9 |
| Bonacci, 2022^78^ | Clinical record/assessment | Incidence of emergency department presentation for endocarditis | 127 | 207 | NR | NR | 3 | 23.6 |
| Bonacci, 2022^78^ | Clinical record/assessment | Incidence of emergency department presentation for bloodstream infection | 127 | 207 | NR | NR | 6 | 47.2 |
| Bonacci, 2022^78^ | Clinical record/assessment | Incidence of emergency department presentation for osteomyelitis | 127 | 207 | NR | NR | 6 | 47.2 |
| Calderon-Villarreal, 2024^13^ | Self-report | Incidence of abscesses | 784 | 647 | 460 (71%) | 43.0  (Median) | NR | 244 |
| Colledge-Frisby, 2022^14^ | ICD codes | Incidence of hospitalisation for skin and soft tissue infection | 454,953 | 47,163 | 31,881 (68%) | 32.0  (Median) | NR | 29.9 |
| Colledge-Frisby, 2022^14^ | ICD codes | Incidence of hospitalisation for endocarditis | 454,953 | 47,163 | 31,881 (68%) | 32.0  (Median) | NR | 2.9 |
| Colledge-Frisby, 2022^14^ | ICD codes | Incidence of hospitalisation for sepsis | 454,953 | 47,163 | 31,881 (68%) | 32.0  (Median) | NR | 7.0 |
| Colledge-Frisby, 2022^14^ | ICD codes | Incidence of hospitalisation for osteomyelitis | 454,953 | 47,163 | 31,881 (68%) | 32.0  (Median) | NR | 3.0 |
| Colledge-Frisby, 2022^14^ | ICD codes | Incidence of hospitalisation for septic arthritis | 454,953 | 47,163 | 31,881 (68%) | 32.0  (Median) | NR | 1.7 |
| Colledge-Frisby, 2022^14^ | ICD codes | Incidence of hospitalisation for any injecting-related infection | 454,953 | 47,163 | 31,881 (68%) | 32.0  (Median) | NR | 42.7 |
| Curtis, 2023^16^ | ICD codes | Incidence of ED presentation for skin and soft tissue infection | 13,106 | 1,288 | 696 (54%) | 32.0  (Median) | 387 | 70 |
| Curtis, 2023^16^ | ICD codes | Incidence of ED presentation for bloodstream infection | 13,106 | 1,288 | 696 (54%) | 32.0  (Median) | 60 | 6 |
| Curtis, 2023^16^ | ICD codes | Incidence of ED presentation for bone or joint infection | 13,106 | 1,288 | 696 (54%) | 32.0  (Median) | 30 | 2 |
| Curtis, 2023^16^ | ICD codes | Incidence of ED presentation for endocarditis | 13,106 | 1,288 | 696 (54%) | 32.0  (Median) | 17 | 1 |
| Curtis, 2023^16^ | ICD codes | Incidence of ED presentation for any injecting-related infection | 13,106 | 1,288 | 696 (54%) | 32.0  (Median) | 427 | 80 |
| Curtis, 2023^16^ | ICD codes | Incidence of hospitalisation for skin and soft tissue infection | 13,106 | 1,288 | 696 (54%) | 32.0  (Median) | 282 | 42 |
| Curtis, 2023^16^ | ICD codes | Incidence of hospitalisation for bloodstream infection | 13,106 | 1,288 | 696 (54%) | 32.0  (Median) | 105 | 12 |
| Curtis, 2023^16^ | ICD codes | Incidence of hospitalisation for bone or joint infection | 13,106 | 1,288 | 696 (54%) | 32.0  (Median) | 48 | 7 |
| Curtis, 2023^16^ | ICD codes | Incidence of hospitalisation for endocarditis | 13,106 | 1,288 | 696 (54%) | 32.0  (Median) | 51 | 6 |
| Curtis, 2023^16^ | ICD codes | Incidence of hospitalisation for any injecting-related infection | 13,106 | 1,288 | 696 (54%) | 32.0  (Median) | 345 | 56 |
| Dahlman, 2018^79^ | ICD codes | Incidence of hospitalisation for skin and soft tissue infection | 27,805 | 2,444 | NR | NR | NR | 28.3 |
| Dahlman, 2018^79^ | ICD codes | Incidence of hospitalisation for cardiac infection | 30,175 | 2,444 | NR | NR | NR | 2.6 |
| Dahlman, 2018^79^ | ICD codes | Incidence of hospitalisation for bone or joint infection | 30,175 | 2,444 | NR | NR | NR | 5.4 |
| Dahlman, 2018^79^ | ICD codes | Incidence of hospitalisation for central nervous system infection | 30,175 | 2,444 | NR | NR | NR | 0.6 |
| Dahlman, 2018^79^ | ICD codes | Incidence of hospitalisation for septicemia | 30,175 | 2,444 | NR | NR | NR | 3.6 |
| Dahlman, 2018^79^ | ICD codes | Incidence of hospitalisation for skin and soft tissue infection, cardiac infection, bone or joint infection, central nervous system infection, or septicemia | 30,175 | 2,444 | NR | NR | NR | 9.1 |
| Dahlman, 2018^79^ | ICD codes | Incidence of mortality due to skin and soft tissue infection, cardiac infection, bone or joint infection, central nervous system infection, or septicemia | 31,196 | 2,444 | NR | NR | NR | 14.7 |
| Figgatt, 2023^80^ | ICD codes | Incidence of mortality due to any injecting-related infection | NR | 131,522 | 54,743 (42%) | 45.0  (Median) | 446 | 1.6 |
| Nambiar, 2017^81^ | ICD codes | Incidence of hospitalisation for skin and soft tissue infection (as primary diagnosis) | 4,163.5 | 2,106 | NR | NR | 75 | 18 |
| Nambiar, 2017^81^ | ICD codes | Incidence of hospitalisation for any injecting-related infection (as primary diagnosis) | 4,163.5 | 2,106 | NR | NR | 89 | 21.4 |
| Nambiar, 2017^81^ | ICD codes | Incidence of hospitalisation for any injecting-related infection (as primary or other diagnosis) | 4,163.5 | 2,106 | NR | NR | 166 | 39.9 |
| Vanichseni, 2015^82^ | Clinical record/assessment | Incidence of mortality due to sepsis | 9,786 | 2,413 | 1,924 (80%) | 31.0  (Median) | 12 | 1.2 |
| Wang, 2023^83^ | ICD codes | Incidence of endocarditis | NR | 736,502 | NR | NR | NR | 30.1 (per 1,000,000 person per day) |

Abbreviations: NR: data not reported; NA: not applicable. *K*= number of studies.

**Supplementary table 11.** Studies evaluating the prevalence of skin and soft tissue infections (not otherwise specified) among people who inject drugs, by prevalence period

| **Study (author, year)** | **Prevalence period** | **Method of infection assessment** | **Infection definition** | **Sample size** | **Males n (%)** | **Age (mean/median)** | **Infection outcome n (%)** | **95% confidence interval (CI)** |
| --- | --- | --- | --- | --- | --- | --- | --- | --- |
| **Lifetime** | | | | | | | | |
| Srivastava, 2023^65^ | Lifetime | ICD codes | Not specified | 216 | 205 (95%) | 53.0  (Mean) | 29 (13%) | 9% - 19% |
| Baltes, 2020^6^ | Lifetime | Self-report | Not specified | 80 | 48 (60%) | NR | 18 (23%) | 15% - 33% |
| Noroozi, 2018^55^ | Lifetime | Self-report | Participants were asked “Have you ever had skin infections (such abscess or cellulitis) at your injection sites?” | 500 | 500 (100%) | 31.2  (Mean) | 200 (40%) | 36% - 44% |
| Dahlman, 2015^17^ | Lifetime | Self-report | Skin and soft tissue infection symptoms were defined as a combination of redness, swelling and pain and/or pus, associated with the injection site. Interviewee was asked “Have you had an abscess or symptoms of skin and soft tissue infection (redness, swelling, pain, pus)?”, and was explicitly asked to distinguish between signs of infection, and irritation caused by extravasal injection. | 80 | 55 (69%) | 44.5 (Median) | 46 (57%) | 46% - 68% |
| Benrubi, 2023^9^ | Lifetime | Self-report | Abscess, ulcer, or cellulitis form at the site of injection | 297 | 150 (51%) | NR | 194 (65%) | 60% - 71% |
| Wright, 2021^74^ | Lifetime | Self-report | Abscess and/or cellulitis. Questions pertaining to experience of skin and soft tissue infection were accompanied by pictures of abscesses and/or cellulitis at different stages. | 284 | NR | NR | 191 (67%) | 62% - 72% |
| Dahlman, 2017^18^ | Lifetime | Self-report | Participants were asked “When did you last have an abscess or symptoms for skin and soft tissue infection (redness, swelling, pain, pus)?” | 201 | 155 (77%) | 44.0  (Median) | 141 (70%) | 63% - 76% |
| **Past 3-12 months** | | | | | | | | |
| Lee, 2013^45^ | Past 6 months | Self-report | Not specified | 430 | 347 (81%) | 38.0  (Median) | 21 (5%) | 3% - 7% |
| Panda, 2014^58^ | Past 12 months | Self-report | Painful skin infection/abscess within the last 1 year | 1,155 | 1,155 (100%) | 27.0  (Median) | 165 (14%) | 12% - 16% |
| NESI, 2020^53^ | Past 12 months | Self-report | Severe skin or soft tissue infection. Participants were asked “Have you had a swelling containing pus (abscess), a sore, or an open wound at an injection site?” | 2,435 | 1,746 (72%) | 40.2  (Median) | 388 (16%) | 15% - 17% |
| NESI, 2016^53^ | Past 12 months | Self-report | Severe skin or soft tissue infection. Participants were asked “Have you had a swelling containing pus (abscess), a sore, or an open wound at an injection site?” | 2,696 | 1,910 (71%) | 37.7  (Median) | 464 (17%) | 16% - 19% |
| NESI, 2023^54^ | Past 12 months | Self-report | Severe skin or soft tissue infection. Participants were asked “Have you had a swelling containing pus (abscess), a sore, or an open wound at an injection site?” | 2,046 | 1,406 (69%) | 43.4  (Median) | 370 (18%) | 16% - 20% |
| NESI, 2018^53^ | Past 12 months | Self-report | Severe skin or soft tissue infection. Participants were asked “Have you had a swelling containing pus (abscess), a sore, or an open wound at an injection site?” | 2,130 | 1,549 (73%) | 40.2  (Median) | 430 (20%) | 19% - 22% |
| Horan, 2019^25^ | Past 3 months | Self-report | Not specified | 51 | 34 (67%) | 30.8  (Mean) | 11 (22%) | 12% - 35% |
| NESI, 2014^53^ | Past 12 months | Self-report | Severe skin or soft tissue infection. Participants were asked “Have you had a swelling containing pus (abscess), a sore, or an open wound at an injection site?” | 2,344 | 1,620 (69%) | 36.7  (Median) | 564 (24%) | 22% - 26% |
| Megerian, 2024^50^ | Past 3 months | Self-report | SSTI were asked as follows: “In the last 3 months, have you had an abscess or other soft tissue infection related to injection drug use?” | 563 | 357 (63%) | NR | 141 (25%) | 22% - 29% |
| Dahlman, 2017^18^ | Past 12 months | Self-report | Participants were asked “When did you last have an abscess or symptoms for skin and soft tissue infection (redness, swelling, pain, pus)?” | 201 | 155 (77%) | 44.0  (Median) | 58 (29%) | 23% - 35% |
| Barocas, 2013^7^ | Past 6 months | Self-report | Not specified | 553 | 368 (69%) | 28  (Median) | 163 (30%) | 26% - 33% |
| Dahlman, 2015^17^ | Past 12 months | Self-report | Skin and soft tissue infection symptoms were defined as a combination of redness, swelling and pain and/or pus, associated with the injection site. Interviewee was asked “Have you had an abscess or symptoms of skin and soft tissue infection (redness, swelling, pain, pus)?”, and was explicitly asked to distinguish between signs of infection, and irritation caused by extravasal injection. | 80 | 55 (69%) | 44.5  (Median) | 24 (30%) | 21% - 41% |
| UAM, 2021^73^ | Past 12 months | Self-report | Symptoms of an injection site infection (abscess, sore or open wound at an injection site) | 803 | NR | NR | 242 (30%) | 27% - 33% |
| Dion, 2020^19^ | Past 12 months | Self-report | Not specified | 141 | 89 (63%) | 37.0  (Mean) | 44 (31%) | 24% - 39% |
| Frost, 2018^21^ | Past 12 months | Self-report | Not specified | 326 | 233 (53%) | 35.5  (Mean) | 108 (33%) | 28% - 38% |
| UAM, 2020^72^ | Past 12 months | Self-report | Symptoms of an injection site infection (abscess, sore or open wound at an injection site) | 485 | NR | NR | 182 (38%) | 33% - 42% |
| UAM, 2019^71^ | Past 12 months | Self-report | Symptoms of an injection site infection (abscess, sore or open wound at an injection site) | 1,913 | NR | NR | 722 (38%) | 36% - 40% |
| Ojha, 2014^56^ | Past 12 months | Self-report | Abscess or skin infection | 300 | 273 (91%) | 28.7  (Mean) | 129 (43%) | 38% - 49% |
| Benrubi, 2023^9^ | Past 12 months | Self-report | Abscess, ulcer, or cellulitis form at the site of injection | 297 | 150 (51%) | NR | 131 (44%) | 39% - 50% |
| UAM, 2017^69^ | Past 12 months | Self-report | Symptoms of an injection site infection (abscess, sore or open wound at an injection site) | 1,483 | NR | NR | 712 (48%) | 45% - 51% |
| Glick, 2021^22^ | Past 12 months | Self-report | Not specified | 720 | 467 (65%) | NR | 385 (54%) | 50% - 57% |
| UAM, 2018^70^ | Past 12 months | Self-report | Symptoms of an injection site infection (abscess, sore or open wound at an injection site) | 1,568 | NR | NR | 845 (54%) | 51% - 56% |
| Jorgensen, 2024^44^ | Past 12 months | Self-report | Not specified | 61 | 35 (57%) | 42.0  (Mean) | 46 (75%) | 63% - 85% |
| Schneider, 2022^63^* | Past 6 months | Self-report | Participants were asked “In the past 6 months, how many times have you had a skin infection or abscess? ” Participants responded numerically, which we then categorized into a binary indicator. Participants who were unsure of how many skin and soft tissue infections they had were considered as having a skin and soft tissue infection. | 114 | 0 | 36.7  (Mean) | 57 (50%) | NA |
| **Past month/current** | | | | | | | | |
| IDRS, 2020^37^ | Past month | Self-report | Participants were asked “Have you had… skin abscess or cellulitis, i.e. a pus-filled lump on your skin (skin abscess), and/or red, hot, swollen and tender/painful skin in an area that is bigger than a 50-cent piece (cellulitis)” | 884 | 522 (59%) | 44.0  (Mean) | 62 (7%) | 6% - 9% |
| IDRS, 2021^38^ | Past month | Self-report | Participants were asked “Have you had… skin abscess or cellulitis, i.e. a pus-filled lump on your skin (skin abscess), and/or red, hot, swollen and tender/painful skin in an area that is bigger than a 50-cent piece (cellulitis)” | 888 | 577 (65%) | 45.0  (Mean) | 62 (7%) | 5% - 9% |
| IDRS, 2023^40^ | Past month | Self-report | Participants were asked “Have you had… skin abscess or cellulitis, i.e. a pus-filled lump on your skin (skin abscess), and/or red, hot, swollen and tender/painful skin in an area that is bigger than a 50-cent piece (cellulitis)” | 817 | 556 (68%) | 46.0  (Median) | 74 (9%) | 7% - 11% |
| IDRS, 2022^39^ | Past month | Self-report | Participants were asked “Have you had… skin abscess or cellulitis, i.e. a pus-filled lump on your skin (skin abscess), and/or red, hot, swollen and tender/painful skin in an area that is bigger than a 50-cent piece (cellulitis)” | 879 | 580 (66%) | 46.0  (Mean) | 87 (10%) | 8% - 12% |
| Dahlman, 2017^18^ | Past month | Self-report | Participants were asked “When did you last have an abscess or symptoms for skin and soft tissue infection (redness, swelling, pain, pus)?” | 201 | 155 (77%) | 44.0  (Median) | 22 (11%) | 7% - 16% |
| IDRS, 2024^41^ | Past month | Self-report | Participants were asked “Have you had… skin abscess or cellulitis, i.e. a pus-filled lump on your skin (skin abscess), and/or red, hot, swollen and tender/painful skin in an area that is bigger than a 50-cent piece (cellulitis)” | 877 | 605 (69%) | 47.0  (Median) | 96 (11%) | 9% - 13% |
| IDRS, 2019^36^ | Past month | Self-report | Participants were asked “Have you had… skin abscess or cellulitis, i.e. a pus-filled lump on your skin (skin abscess), and/or red, hot, swollen and tender/painful skin in an area that is bigger than a 50-cent piece (cellulitis)” | 902 | 613 (68%) | 44.0  (Mean) | 104 (12%) | 10% - 14% |
| Dahlman, 2015^17^ | Past month | Self-report | Skin and soft tissue infection symptoms were defined as a combination of redness, swelling and pain and/or pus, associated with the injection site. The interviewee was asked “Have you had an abscess or symptoms of skin and soft tissue infection (redness, swelling, pain, pus)?”, and was explicitly asked to distinguish between signs of infection, and irritation caused by extravascular injection. | 65 | NR | NR | 9 (14%) | 7% - 25% |
| Aslam, 2022^4^ | Current | Clinical record/  Assessment | Not specified | 217 | NR | NR | 41 (19%) | 14% - 25% |
| Benrubi, 2023^9^ | Past month | Self-report | Abscess, ulcer, or cellulitis form at the site of injection | 297 | 150 (51%) | NR | 60 (20%) | 16% - 25% |
| Perri, 2021^59^ | Past month | Self-report | Not specified | 56 | NR | NR | 30 (54%) | 41% - 66% |

Abbreviations: NR: data not reported; NA: not applicable; IBBS: Integrated Biological Behavioural Surveillance; NESI: Needle Exchange Surveillance Initiative; UAM: Unlinked Anonymous Monitoring Survey

*Study not included in meta-analysis

**Supplementary table 12.** Studies evaluating the prevalence of skin abscess among people who inject drugs, by prevalence period

| **Study (author, year)** | **Prevalence period** | **Method of infection assessment** | **Infection definition** | **Sample size** | **Males n (%)** | **Age (mean/median)** | **Infection outcome n (%)** | **95% confidence interval (CI)** |
| --- | --- | --- | --- | --- | --- | --- | --- | --- |
| **Lifetime** | | | | | | | | |
| IBBS, 2018^26^ | Lifetime | Self-report | Not specified | 500 | 431 (86%) | NR | 29 (6%) | 4% - 8% |
| Yen, 2014^76^ | Lifetime | Self-report | Cutaneous abscess | 827 | 710 (86%) | 45.0  (Median) | 115 (14%) | 12% - 16% |
| IDRS, 2012^29^ | Lifetime | Self-report | Skin abscess (pus-filled lump) | 924 | 610 (66%) | 39.0  (Mean) | 175 (19%) | 17% - 22% |
| Ivan, 2016^42^ | Lifetime | Self-report | Not specified | 45 | NR | NR | 12 (27%) | 16% - 41% |
| Bicket, 2020^10^ | Lifetime | Self-report | Not specified | 203 | 132 (65%) | NR | 72 (36%) | 29% - 42% |
| Wurcel, 2018^75^ | Lifetime | Self-report | Participants were asked "Has a medical professional ever told you that you had an abscess?" | 298 | 210 (71%) | 33.0  (Median) | 125 (42%) | 36% - 48% |
| Marks, 2024^48^ | Lifetime | Self-report | Abscess or ‘boil’ at an injection site | 728 | 464 (64%) | NR | 306 (42%) | 38% - 46% |
| Coull, 2021^15^ | Lifetime | Self-report | Raised red hot painful lumps, with or without obvious pus/broken skin – possibly required lancing/surgery or have spontaneously burst | 128 | 97 (76%) | NR | 58 (45%) | 37% - 54% |
| Syvertsen, 2014^67^ | Lifetime | Self-report | Not specified | 150 | 127 (85%) | 28.7  (Mean) | 68 (46%) | 38% - 53% |
| Asher, 2019^3^ | Lifetime | Self-report | Cutaneous abscess caused by bacterial infection | 541 | 388 (72%) | NR | 258 (48%) | 44% - 52% |
| Ambekar, 2015^2^ | Lifetime | Self-report | Not specified | 902 | 902 (100%) | 33.4  (Mean) | 507 (56%) | 53% - 59% |
| Summers, 2018^66^ | Lifetime | Self-report | Not specified | 145 | 103 (71%) | 40.0  (Mean) | 85 (59%) | 50% - 66% |
| Ozga, 2021^57^ | Lifetime | Self-report | Not specified | 494 | 299 (61%) | 46.0  (Median) | 332 (67%) | 63% - 71% |
| Maloney, 2010^47^ | Lifetime | Self-report | Not specified | 70 | NR | NR | 48 (69%) | 57% - 78% |
| Li, 2021^46^ | Lifetime | Clinical record/  assessment | Not specified | 48 | 39 (81%) | NR | 36 (75%) | 61% - 85% |
| **Past 3-12 months** | | | | | | | | |
| Heimer, 2015^24^ | Past 12 months | Self-report | Not specified | 390 | 373 (96%) | 28.0  (Median) | 6 (2%) | 1% - 3% |
| IDRS, 2012^29^ | Past 6 months | Self-report | Skin abscess (pus-filled lump) | 924 | 610 (66%) | 39.0  (Mean) | 59 (6%) | 5% - 8% |
| Ivan, 2016^42^ | Past 12 months | Self-report | Not specified | 45 | NR | NR | 5 (11%) | 5% - 24% |
| Roux, 2022^62^ | Past 6 months | Self-report | Not specified | 662 | 529 (80%) | 39.0  (Median) | 135 (20%) | 17% - 24% |
| Calderon-Villarreal, 2024^13^ | Past 6 months | Self-report | Having at least one abscess (‘cuerazo’ in Spanish slang) at a location on their bodies where they inject drugs in the last six months | 647 | 460 (71%) | 43.0  (Median) | 136 (21%) | 18% - 24% |
| Ozga, 2021^57^ | Past 3 months | Self-report | Not specified | 494 | 299 (61%) | 46.0  (Median) | 112 (23%) | 19% - 27% |
| Roux, 2021^61^ | Past 6 months | Self-report | Cutaneous abscess | 307 | 254 (83%) | 38.0  (Median) | 76 (25%) | 20% - 30% |
| Frank, 2024^20^ | Past 12 months | Self-report | Not specified | 822 | 476 (58%) | NR | 275 (33%) | 30% - 37% |
| Goncalves, 2023^23^ | Past 12 months | Self-report | Cutaneous abscess | 140 | 109 (78%) | 39.0  (Median) | 49 (35%) | 28% - 43% |
| Frost, 2018^21^ | Past 12 months | Self-report | Not specified | 436 | 233 (53%) | 35.5  (Mean) | 173 (40%) | 35% - 44% |
| **Past month/current** | | | | | | | | |
| Wurcel, 2018^75^ | Past month | Self-report | Participants were asked "Has a medical professional ever told you that you had an abscess?" | 298 | 210 (71%) | 33.0  (Median) | 6 (2%) | 1% - 4% |
| Ivan, 2016^42^ | Past month | Self-report | Not specified | 45 | NR | NR | 4 (9%) | 3% - 21% |
| Asher, 2019^3^ | Current | Self-report | Cutaneous abscess caused by bacterial infection | 541 | 388 (72%) | NR | 53 (10%) | 8% - 13% |
| Johnson, 2013^43^ | Current | Self-report | Not specified | 81 | NR | NR | 14 (17%) | 11% - 27% |
| Smith, 2015^64^ | Current | Clinical record/  assessment | Abscesses were defined as swollen, red, painful lumps under the skin that may or may not be open and that have lasted <8 weeks. Study team members visually verified the presence of current abscesses and wounds at the time of the survey. | 152 | 96 (63%) | 45.0  (Median) | 27 (18%) | 12% - 25% |
| Perri, 2021^59^ | Past month | Self-report | Not specified | 56 | NR | NR | 11 (20%) | 11% - 32% |

Abbreviations: NR: data not reported; NA: not applicable; IBBS: Integrated Biological Behavioural Surveillance; IDRS: Illicit Drug Reporting System

*Study not included in meta-analysis

**Supplementary table 13.** Studies evaluating the prevalence of cellulitis among people who inject drugs, by prevalence period

| **Study** | **Prevalence period** | **Method of infection assessment** | **Infection definition** | **Sample size** | **Number of males (%)** | **Mean or median age** | **Number with infection (%)** | **95% confidence interval (CI)** |
| --- | --- | --- | --- | --- | --- | --- | --- | --- |
| **Lifetime** | | | | | | | | |
| IDRS, 2012^29^ | Lifetime | Self-report | Red, hot, swollen, tender skin | 924 | 610 (66%) | 39.0  (Mean) | 223 (24%) | 21%- 27% |
| Ivan, 2016^42^ | Lifetime | Self-report | Not specified | 45 | NR | NR | 11 (24%) | 13% - 38% |
| Marks, 2024^48^ | Lifetime | Self-report | Skin redness at an injection site | 728 | 464 (64%) | NR | 358 (49%) | 46% - 53% |
| **Past 3-12 months** | | | | | | | | |
| Ivan, 2016^42^ | Past 12 months | Self-report | Not specified | 45 | NR | NR | 4 (9%) | 2% - 19% |
| IDRS, 2012^29^ | Past 6 months | Self-report | Red, hot, swollen, tender skin | 924 | 610 (66%) | 39.0  (Mean) | 102 (11%) | 9% - 13% |
| **Past month/current** | | | | | | | | |
| Ivan, 2016^42^* | Past month | Self-report | Not specified | 45 | NR | NR | 2 (4%) | NA |

Abbreviations: NR: data not reported; NA: not applicable; IDRS: Illicit Drug Reporting System

*Study not included in meta-analysis

**Supplementary figure 1**. Forest plot of the prevalence of cellulitis among people who inject drugs, by prevalence period

**Supplementary table 14.** Pooled estimates of recent and lifetime prevalence of injecting-related infection among people who inject drugs, by country-level needle-syringe program (NSP) and opioid agonist treatment (OAT) coverage

| **Outcome** | **Total number of estimates** | **Overall prevalence estimate (95% CI)** | **Prevalence by NSP coverage** | | | | **Prevalence by OAT coverage** | | | | **Prevalence by NSP-OAT coverage** | | | |
| --- | --- | --- | --- | --- | --- | --- | --- | --- | --- | --- | --- | --- | --- | --- |
|  |  |  | **High** | | **Low-moderate** | | **High** | | **Low-moderate** | | **High** | | **Low-moderate** | |
|  |  |  | **Number of estimates** | **Prevalence estimate (95% CI)** | **Number of estimates** | **Prevalence estimate (95% CI)** | **Number of estimates** | **Prevalence estimate (95% CI)** | **Number of estimates** | **Prevalence estimate (95% CI)** | **Number of estimates** | **Prevalence estimate (95% CI)** | **Number of estimates** | **Prevalence estimate (95% CI)** |
| **Skin and soft tissue infection** | | | | | |  |  |  |  |  |  |  |  |  |
| Lifetime^ | 7 | 47%  (29-66%) | 0 | - | 7 | 47%  (29-66%) | 3 | 55%  (38-70%) | 4 | 40%  (15-72%) | 0 | - | 7 | 47%  (29-66%) |
| Recent^ | 34 | 24%  (18-30%) | 6 | 9% (8-11%) | 28 | 28%  (23-35%) | 15 | 17%  (11-24%) | 19 | 30%  (23-38%) | 6 | 9% (8-11%) | 28 | 28% (23-35%) |
| **Abscess** | | | | | |  |  |  |  |  |  |  |  |  |
| Lifetime^ | 15 | 41%  (29-54%) | 2 | 21%  (15-27%) | 13 | 44%  (31-58%) | 5 | 28%  (11-56%) | 10 | 47%  (35-59%) | 2 | 21%  (15-27%) | 13 | 44%  (31-58%) |
| Recent^ | 15 | 17%  (12-24%) | 3 | 7%  (5-10%) | 12 | 17%  (10-26%) | 5 | 15%  (7-27%) | 10 | 15%  (8-26%) | 3 | 7%  (5-10%) | 12 | 17%  (10-26%) |
| **Endocarditis** | | | | | |  |  |  |  |  |  |  |  |  |
| Lifetime^ | 8 | 6%  (3-10%) | 2 | 6%  (2-16%) | 6 | 6%  (3-11%) | 4 | 5%  (1-18%) | 4 | 5%  (3-9%) | 2 | 6%  (2-16%) | 6 | 6%  (3-11%) |
| Recent^ | 9 | 2%  (1-3%) | 5 | 2%  (1-3%) | 4 | 2%  (2-3%) | 5 | 2%  (1-3%) | 4 | 2%  (2-3%) | 5 | 2%  (1-3%) | 4 | 2%  (2-3%) |

^Lifetime refers to infection ever, but not in the last 12 months; recent refers to infection in the last 12 months (e.g., composite of current, last 1 month, and last 3-12 months)

**Supplementary table 15.** Pooled estimates of recent and lifetime prevalence of injecting-related infection among people who inject drugs, by income level in country of estimate

| **Outcome** | **Total number of estimates** | **Overall prevalence estimate (95% CI)** | **Number of LMIC estimates** | **LMIC prevalence estimate (95% CI)** | **Number of HIC estimates** | **HIC prevalence estimate (95% CI)** |
| --- | --- | --- | --- | --- | --- | --- |
| **Skin and soft tissue infection** | | | | | | |
| Lifetime^ | 7 | 47% (29-66%) | 1 | - | 6 | 48% (27-70%) |
| Recent^ | 34 | 24% (18-30%) | 4 | 17% (6-37%) | 30 | 25% (19-31%) |
| **Abscess** | | | | | | |
| Lifetime^ | 15 | 41% (29-54%) | 3 | 29% (6-72%) | 12 | 44% (32-56%) |
| Recent^ | 15 | 17% (12-24%) | 1 | - | 14 | 17% (12-24%) |

^Lifetime refers to infection ever, but not in the last 12 months; recent refers to infection in the last 12 months (e.g., composite of current, last 1 month, and last 3-12 months)

Abbreviation: HIC, high income country; LMIC, low middle income country

**Supplementary table 16.** Pooled estimates of recent and lifetime prevalence of injecting-related infection among people who inject drugs, by country of estimate

| **Outcome** | **Total number of estimates** | **Overall prevalence estimate (95% CI)** | **Country** | **Number of estimates** | **Country-level prevalence estimate (95% CI)** |
| --- | --- | --- | --- | --- | --- |
| **Skin and soft tissue infection** | | | | | |
| Lifetime^ | 7 | 47% (29-66%) | USA | 4 | 40% (15-72%) |
| Recent^ | 34 | 24% (18-30%) | Australia India  Scotland  UK*  USA | 6  2  5  5  10 | 9% (8-11%)  16% (12-21%)  19% (16-22%)  41% (33-50%)  32% (23-41%) |
| **Abscess** | | | | | |
| Lifetime^ | 15 | 41% (29-54%) | Australia  USA | 2  7 | 21% (15-27%)  52% (41-63%) |
| Recent^ | 15 | 17% (12-24%) | Australia  USA  France | 3  8  2 | 7% (5-10%)  17% (9-29%)  27% (15-43%) |
| **Endocarditis** | | | | | |
| Lifetime^ | 8 | 6% (3-10%) | USA | 4 | 5% (1-18%) |
| Recent^ | 9 | 2% (1-3%) | Australia  USA | 5  4 | 2% (1-3%)  2% (2-3%) |

^Lifetime refers to infection ever, but not in the last 12 months; recent refers to infection in the last 12 months (e.g., composite of current, last 1 month, and last 3-12 months)

*England, Wales, and Northern Ireland

Overall prevalence
Past 12 months, 30%

**Supplementary figure 2.** Estimates of recent (past 12 months) skin and soft tissue infection by country-level needle-syringe program (NSP) and opioid agonist treatment (OAT) coverage

Overall prevalence
Past 12 months, 30%

**Supplementary figure 3.** Estimates of recent (past 12 months) skin and soft tissue infection by country

**Supplementary table 17.** Studies evaluating the prevalence of hospitalisation for injecting-related infection

| **Study** | **Prevalence period** | **Method of infection assessment** | **Infection definition** | **Sample size** | **Number of males (%)** | **Mean or median age** | **Number hospitalised (%)** | **95% confidence interval (CI)** |
| --- | --- | --- | --- | --- | --- | --- | --- | --- |
| Colledge-Frisby, 2022^14^ | Lifetime | ICD codes | Hospitalisation with skin and soft tissue infection | 47,163 | 31,881 (68%) | 32.0  (Median) | 6,973 (15%) | NA |
| Colledge-Frisby, 2022^14^ | Lifetime | ICD codes | Hospitalisation with endocarditis | 47,163 | 31,881 (68%) | 32.0  (Median) | 791 (2%) | NA |
| Colledge-Frisby, 2022^14^ | Lifetime | ICD codes | Hospitalisation with sepsis | 47,163 | 31,881 (68%) | 32.0  (Median) | 2,386 (5%) | NA |
| Colledge-Frisby, 2022^14^ | Lifetime | ICD codes | Hospitalisation with osteomyelitis | 47,163 | 31,881 (68%) | 32.0  (Median) | 781 (2%) | NA |
| Colledge-Frisby, 2022^14^ | Lifetime | ICD codes | Hospitalisation with septic arthritis | 47,163 | 31,881 (68%) | 32.0  (Median) | 543 (1%) | NA |
| Colledge-Frisby, 2022^14^ | Lifetime | ICD codes | Hospitalisation with any injecting-related infection | 47,163 | 31,881 (68%) | 32.0  (Median) | 9,122 (19%) | NA |
| Curtis, 2023^16^ | Lifetime | ICD codes | Hospitalisation with skin and soft tissue infection | 1288 | 696 (54%) | 32.0  (Median) | 282 (22%) | NA |
| Curtis, 2023^16^ | Lifetime | ICD codes | Hospitalisation with bloodstream infection | 1288 | 696 (54%) | 32.0  (Median) | 105 (8%) | NA |
| Curtis, 2023^16^ | Lifetime | ICD codes | Hospitalisation with bone or joint infection (osteomyelitis or septic arthritis) | 1288 | 696 (54%) | 32.0  (Median) | 48 (4%) | NA |
| Curtis, 2023^16^ | Lifetime | ICD codes | Hospitalisation with endocarditis | 1288 | 696 (54%) | 32.0  (Median) | 51 (4%) | NA |
| Curtis, 2023^16^ | Lifetime | ICD codes | Hospitalisation with any injecting-related infection | 1288 | 696 (54%) | 32.0  (Median) | 345 (27%) | NA |
| Dion, 2020^19^ | Past 12 months | Self-report | Hospitalisation with endocarditis | 141 | 89 (63%) | 37.0  (Mean) | 6 (4%) | NA |
| Evans, 2018^84^ | Lifetime | ICD codes | Hospitalisation with arm cellulitis or abscess (ICD-9-CM 682.3) | 20,759 | 9,493 (46%) | 37.0  (Mean) | 982 (5%) | NA |
| IDRS, 2012^29^ | Lifetime | Self-report | Any serious injecting-related infection requiring hospitalisation and intravenous antibiotics | 924 | 610 (66%) | 39.0  (Mean) | 124 (13%) | NA |
| IDRS, 2012^29^ | Past 6 months | Self-report | Any serious injecting-related infection requiring hospitalisation and intravenous antibiotics | 924 | 610 (66%) | 39.0  (Mean) | 33 (4%) | NA |
| Ivan, 2016^42^ | Lifetime | Self-report | Hospitalisation with septicemia | 45 | NR | NR | 3 (7%) | NA |
| Jorgensen, 2024^44^ | Past 12 months | Self-report | Hospitalisation with any injecting-related infection | 61 | 35 (57%) | 42.0  (Mean) | 5 (8%) | NA |
| Ozga, 2023^57^ | Past 3 months | Self-report | Hospitalisation with a skin abscess | 494 | 299 (61%) | 46.0  (Median) | 7 (1%) | NA |
| Wheeler, 2022^85^ | Past 12 months | Self-report | Hospitalisation with abscess or cellulitis | 1,851 | 1,241 (67%) | 43.0  (Median) | 377 (20%) | NA |

**Supplementary table 18.** Studies evaluating the prevalence of endocarditis among people who inject drugs, by prevalence period

| **Study** | **Prevalence period** | **Method of infection assessment** | **Infection definition** | **Sample size** | **Number of males (%)** | **Mean or median age** | **Number with infection (%)** | **95% confidence interval (CI)** |
| --- | --- | --- | --- | --- | --- | --- | --- | --- |
| **Lifetime** | | | | | | | | |
| Srivastava, 2023^65^ | Lifetime | ICD codes | Not specified | 216 | 205 (95%) | 53.0  (Mean) | 1 (1%) | 1% - 3% |
| IDRS, 2012^29^ | Lifetime | Self-report | An infection in the heart, where you need to stay in hospital and have (up to 6 weeks of) regular intravenous antibiotics | 924 | 610 (66%) | 39.0  (Mean) | 32 (4%) | 2% - 5% |
| Zimmerman, 2016^77^ | Lifetime | Self-report | Not specified | 2,077 | 1,594 (77%) | 38.0  (Median) | 73 (4%) | 3% - 4% |
| Marks, 2024^48^ | Lifetime | Self-report | Infection of the heart valve | 728 | 464 (64%) | NR | 31 (4%) | 3% - 6% |
| Axelsson, 2014^5^ | Lifetime | Self-report | Not specified | 206 | 159 (77%) | 43.0  (Mean) | 14 (7%) | 4% - 11% |
| Bicket, 2020^10^ | Lifetime | Self-report | Not specified | 203 | 132 (65%) | NR | 18 (9%) | 5% - 13% |
| Morin, 2020^52^ | Lifetime | Clinical record/ assessment | Pathogenic infection of the endocardial tissue and heart valves | 55,924 | 3,6229 (65%) | NR | 5,642 (10%) | 10% - 10% |
| Li, 2021^46^ | Lifetime | Clinical record/ assessment | Not specified | 48 | 39 (81%) | NR | 9 (19%) | 9% - 31% |
| **Past 3-12 months** | | | | | | | | |
| Barocas, 2013^7^ | Past 6 months | Self-report | Not specified | 553 | 368 (69%) | 28  (Median) | 7 (1%) | 1% - 2% |
| IDRS, 2012^29^ | Past 6 months | Self-report | Not specified | 924 | 610 (66%) | 39.0  (Mean) | 16 (2%) | 1% - 3% |
| Frank, 2024^20^ | Past 12 months | Self-report | Not specified | 822 | 476 (58%) | NR | 16 (2%) | 1% - 3% |
| Glick, 2021^22^ | Past 12 months | Self-report | Not specified | 720 | 467 (65%) | NR | 17 (2%) | 1% - 2% |
| Megerian, 2024^50^ | Past 3 months | Self-report | Endocarditis was asked as follows: “In the last 3 months has a doctor, nurse or counselor told you that you have endocarditis (i.e., an infection in your heart valve)?” | 563 | 357 (63%) | NR | 16 (3%) | 2% - 5% |
| **Past month/current** | | | | | | | | |
| IDRS, 2021^38^ | Past month | Self-report | An infection in the heart, where you need to stay in hospital and have (up to 6 weeks of) regular intravenous antibiotics | 888 | 577 (65%) | 45.0  (Mean) | 9 (1%) | 1% - 2% |
| IDRS, 2024^41^ | Past month | Self-report | An infection in the heart, where you need to stay in hospital and have (up to 6 weeks of) regular intravenous antibiotics | 877 | 605 (69%) | 47.0  (Median) | 9 (1%) | 1% - 2% |
| IDRS, 2019^36^ | Past month | Self-report | An infection in the heart, where you need to stay in hospital and have (up to 6 weeks of) regular intravenous antibiotics | 902 | 613 (68%) | 44.0  (Mean) | 26 (3%) | 2% - 4% |
| IDRS, 2022^39^ | Past month | Self-report | An infection in the heart, where you need to stay in hospital and have (up to 6 weeks of) regular intravenous antibiotics | 879 | 580 (66%) | 46.0  (Mean) | 26 (3%) | 2% - 4% |

Abbreviations: NR: data not reported; NA: not applicable; IDRS: Illicit Drug Reporting System

*Study not included in meta-analysis

**Supplementary table 19.** Studies evaluating the prevalence of bloodstream infection and/or sepsis among people who inject drugs, by prevalence period

| **Study** | **Prevalence period** | **Method of infection assessment** | **Infection definition** | **Sample size** | **Number of males (%)** | **Mean or median age** | **Number with infection (%)** | **95% confidence interval (CI)** |
| --- | --- | --- | --- | --- | --- | --- | --- | --- |
| **Lifetime** | | | | | | | | |
| Srivastava, 2023^65^ | Lifetime | ICD codes | Not specified | 216 | 205 (95%) | 53.0  (Mean) | 3 (1%) | 1% - 4% |
| Ivan, 2016^42^ | Lifetime | Self-report | Septicaemia | 45 | NR | NR | 3 (7%) | 2% - 19% |
| Bicket, 2020^10^ | Lifetime | Self-report | Septicaemia | 203 | 132 (65%) | NR | 14 (7%) | 4% - 11% |
| Marks, 2024^48^ | Lifetime | Self-report | Bloodstream infection or sepsis | 728 | 464 (64%) | NR | 74 (10%) | 8% - 13% |
| Li, 2021^46^ | Lifetime | Clinical record/  assessment | Bacteraemia | 48 | 39 (81%) | NR | 13 (27%) | 16% - 41% |
| **Past 3-12 months** | | | | | | | | |
| Ivan, 2016^42^ | Past 12 months | Self-report | Septicaemia | 45 | NR | NR | 2 (4%) | 1% - 16% |
| Frank, 2024^20^ | Past 12 months | Self-report | Bloodstream infection or sepsis | 822 | 476 (58%) | NR | 46 (6%) | 4% - 7% |
| Glick, 2021^22^ | Past 12 months | Self-report | Infected blood clot or blood infection | 720 | 467 (65%) | NR | 78 (11%) | 9% - 13% |
| **Past month/current** | | | | | | | | |
| IDRS, 2019^36^ | Past month | Self-report | Sepsis | 902 | 613 (68%) | 44.0  (Mean) | 9 (1%) | 1% - 2% |
| Ivan, 2016^42^ | Past month | Self-report | Septicaemia | 45 | NR | NR | 1 (2%) | 0% - 14% |

Abbreviations: NR: data not reported; NA: not applicable; IDRS: Illicit Drug Reporting System

*Study not included in meta-analysis

**Supplementary figure 4**. Forest plot of the prevalence of bloodstream infection and/or sepsis among people who inject drugs, by prevalence period

**Supplementary table 20.** Studies evaluating the prevalence of osteomyelitis among people who inject drugs, by prevalence period

| **Study** | **Prevalence period** | **Method of infection assessment** | **Infection definition** | **Sample size** | **Number of males (%)** | **Mean or median age** | **Number with infection (%)** | **95% confidence interval (CI)** |
| --- | --- | --- | --- | --- | --- | --- | --- | --- |
| **Lifetime** | | | | | | | | |
| Morin, 2020^52^ | Lifetime | Clinical record/ assessment | Not specified | 55,924 | 3,6229 (65%) | NR | 1,236 (2%) | 2% - 2% |
| Srivastava, 2023^65^ | Lifetime | ICD codes | Not specified | 216 | 205 (95%) | 53.0  (Mean) | 5 (2%) | 1% - 5% |
| Li, 2021^46^ | Lifetime | Clinical record/ assessment | Not specified | 48 | 39 (81%) | NR | 6 (12%) | 6% - 25% |
| **Past month/current** | | | | | | | | |
| IDRS, 2019^36^* | Past month | Self-report | Not specified | 902 | 613 (68%) | 44.0  (Mean) | 9 (1%) | NA |

Abbreviations: NR: data not reported; NA: not applicable; IDRS: Illicit Drug Reporting System

*Study not included in meta-analysis

**Supplementary table 21.** Studies evaluating the prevalence of septic arthritis among people who inject drugs, by prevalence period

| **Study** | **Prevalence period** | **Method of infection assessment** | **Infection definition** | **Sample size** | **Number of males (%)** | **Mean or median age** | **Number with infection (%)** | **95% confidence interval (CI)** |
| --- | --- | --- | --- | --- | --- | --- | --- | --- |
| **Lifetime** | | | | | | | | |
| Srivastava, 2023^65^ | Lifetime | ICD codes | Not specified | 216 | 205 (95%) | 53.0  (Mean) | 1 (<1%) | 1% - 3% |
| Morin, 2020^52^ | Lifetime | Clinical record/ assessment | Not specified | 55,924 | 3,6229 (65%) | NR | 576 (1%) | 1% - 1% |
| Li, 2021^46^ | Lifetime | Clinical record/ assessment | Not specified | 48 | 39 (81%) | NR | 4 (8%) | 3% - 20% |
| **Past month/current** | | | | | | | | |
| IDRS, 2019^36^* | Past month | Self-report | Not specified | 902 | 613 (68%) | 44.0  (Mean) | 9 (1%) | NA |

Abbreviations: NR: data not reported; NA: not applicable; IDRS: Illicit Drug Reporting System

*Study not included in meta-analysis

**Supplementary table 22.** Studies evaluating the prevalence of multiple, any, and other injecting-related infection among people who inject drugs, by prevalence period

| **Study** | **Prevalence period** | **Method of infection assessment** | **Infection definition** | **Sample size** | **Number of males (%)** | **Mean or median age** | **Number with infection (%)** | **95% confidence interval (CI)** |
| --- | --- | --- | --- | --- | --- | --- | --- | --- |
| **Lifetime** | | | | | | | | |
| Alexander, 2022^1^* | Lifetime | Self-report | Endocarditis or skin and soft tissue infection | 77 | NR | NR | 42 (54%) | NA |
| Batisse, 2022^8^ | Lifetime | Clinical record/  assessment | Any injecting-related infection (‘Bacterial complications related to injecting’) | 186 | NR | NR | 10 (5%) | NA |
| Bull-Otterson, 2020^12^* | Lifetime | ICD codes | Abscess, cellulitis, acute osteomyelitis, or endocarditis | 844,242 | 430,827 (51%) | NR | 547,780 (65%) | NA |
| IDRS, 2012^29^* | Lifetime | Self-report | Internal abscess | 924 | 610 (66%) | 39.0  (Mean) | 82 (9%) | NA |
| IDRS, 2012^29^* | Lifetime | Self-report | Thrombophlebitis | 924 | 610 (66%) | 39.0  (Mean) | 231 (25%) | NA |
| IDRS, 2012^29^* | Lifetime | Self-report | Gangrene | 924 | 610 (66%) | 39.0  (Mean) | 40 (4%) | NA |
| Li, 2021^46^* | Lifetime | Clinical record/ assessment | Paraspinal abscess | 48 | 39 (81%) | NR | 2 (4%) | NA |
| Marks, 2024^48^ | Lifetime | Self-report | Cellulitis, abscess, open wound at injection site, bloodstream infection or sepsis, endocarditis | 728 | 464 (64%) | NR | 462 (63%) | NA |
| Mezaache, 2021^51^* | Lifetime | Self-report | Cutaneous abscess or sepsis | 552 | 445 (80%) | 4.0  (Median) | 255 (46%) | NA |
| Morin, 2020^52^* | Lifetime | Clinical record/ assessment | Endocarditis, osteomyelitis, or septic arthritis | 55,924 | 3,6229 (65%) | NR | 6,784 (12%) | NA |
| Peyriere, 2013^60^* | Lifetime | Clinical record/ assessment | Local pain, erythema and/or abscess, endocarditis, spondylodiscitis, sepsis | 383 | NR | NR | 25 (7%) | NA |
| Srivastava, 2023^65^ | Lifetime | ICD codes | Epidural abscess | 216 | 205 (95%) | 53.0  (Mean) | 1 (<1%) | NA |
| **Past 3-12 months** | | | | | | | | |
| IDRS, 2012^29^* | Past 6 months | Self-report | Internal abscess | 924 | 610 (66%) | 39.0  (Mean) | 25 (3%) | NA |
| IDRS, 2012^29^* | Past 6 months | Self-report | Thrombophlebitis | 924 | 610 (66%) | 39.0  (Mean) | 151 (16%) | NA |
| IDRS, 2012^29^* | Past 6 months | Self-report | Gangrene | 924 | 610 (66%) | 39.0  (Mean) | 15 (2%) | NA |
| McMahan, 2020^49^* | Past 12 months | Self-report | Past-year infection that was likely related to injection (i.e., an abscess, skin infection such as cellulitis, blood clot or blood infection like sepsis, or endocarditis). | 583 | 322 (55%) | 35.0  (Median) | 250 (43%) | NA |
| Taylor, 2019^68^* | Past 6 months | Self-report | Any injecting-related infection | 72 | 58 (81%) | 43.5  (Mean) | 21 (29%) | NA |
| **Past month/current** | | | | | | | | |
| Bonar, 2011^11^* | Current | Self-report | Any injecting-related infection | 91 | 70 (77%) | 45.3  (Mean) | 6 (7%) | NA |
| IDRS, 2010^27^* | Past month | Self-report | Any injecting-related infection | 902 | 586 (65%) | 38.0  (Mean) | 81 (9%) | NA |
| IDRS, 2011^28^* | Past month | Self-report | Any injecting-related infection | 868 | 573 (66%) | 39.0  (Mean) | 74 (9%) | NA |
| IDRS, 2012^29^* | Past month | Self-report | Any injecting-related infection | 924 | 610 (66%) | 39.0  (Mean) | 76 (8%) | NA |
| IDRS, 2013^30^* | Past month | Self-report | Any injecting-related infection | 887 | 568 (64%) | 40.0  (Mean) | 43 (5%) | NA |
| IDRS, 2014^31^* | Past month | Self-report | Any injecting-related infection | 898 | 620 (69%) | 41.0  (Mean) | 41 (5%) | NA |
| IDRS, 2015^32^* | Past month | Self-report | Any injecting-related infection | 888 | 595 (67%) | 42.0  (Mean) | 49 (6%) | NA |
| IDRS, 2016^33^* | Past month | Self-report | Any injecting-related infection | 877 | 605 (69%) | 43.0  (Mean) | 68 (8%) | NA |
| IDRS, 2017^34^* | Past month | Self-report | Any injecting-related infection | 888 | 595 (67%) | 43.0  (Mean) | 61 (7%) | NA |
| IDRS, 2018^35^* | Past month | Self-report | Any injecting-related infection | 905 | 597 (66%) | 43.0  (Mean) | 66 (7%) | NA |
| IDRS, 2019^36^* | Past month | Self-report | Any injecting-related infection | 902 | 613 (68%) | 44.0  (Mean) | 121 (13%) | NA |
| IDRS, 2020^37^* | Past month | Self-report | Any injecting-related infection | 884 | 522 (59%) | 44.0  (Mean) | 70 (8%) | NA |
| IDRS, 2021^38^* | Past month | Self-report | Any injecting-related infection | 888 | 577 (65%) | 45.0  (Mean) | 71 (8%) | NA |
| IDRS, 2022^39^* | Past month | Self-report | Any injecting-related infection | 879 | 580 (66%) | 46.0  (Mean) | 105 (12%) | NA |
| IDRS, 2023^40^ | Past month | Self-report | Any injecting-related infection | 817 | 556 (68%) | 46.0  (Median) | 82 (10%) | NA |
| IDRS, 2024^41^ | Past month | Self-report | Any injecting-related infection | 877 | 605 (69%) | 47.0  (Median) | 114 (13%) | NA |

Abbreviations: NR: data not reported; NA: not applicable; IDRS: Illicit Drug Reporting System

*Study not included in meta-analysis

**Supplementary table 23.** Studies evaluating the prevalence of emergency department presentations for injecting-related infection

| **Study** | **Prevalence period** | **Method of infection assessment** | **Infection definition** | **Sample size** | **Number of males (%)** | **Mean or median age** | **Number who presented to ED (%)** | **95% confidence interval (CI)** |
| --- | --- | --- | --- | --- | --- | --- | --- | --- |
| Curtis, 2023^16^ | Lifetime | ICD codes | Emergency department presentation with skin and soft tissue infection | 1288 | 696 (54%) | 32.0  (Median) | 387 (30%) | NA |
| Curtis, 2023^16^ | Lifetime | ICD codes | Emergency department presentation with bloodstream infection | 1288 | 696 (54%) | 32.0  (Median) | 60 (5%) | NA |
| Curtis, 2023^16^ | Lifetime | ICD codes | Emergency department presentation with bone or joint infection (osteomyelitis or septic arthritis) | 1288 | 696 (54%) | 32.0  (Median) | 30 (2%) | NA |
| Curtis, 2023^16^ | Lifetime | ICD codes | Emergency department presentation with endocarditis | 1288 | 696 (54%) | 32.0  (Median) | 17 (1%) | NA |
| Curtis, 2023^16^ | Lifetime | ICD codes | Emergency department presentation with any injecting-related infection | 1288 | 696 (54%) | 32.0  (Median) | 427 (33%) | NA |
| Ozga, 2023^57^ | Past 3 months | Self-report | Emergency department presentation for an abscess | 494 | 299 (61%) | 46.0  (Median) | 20 (4%) | NA |
| Schneider, 2022^63^ | Past 6 months | Self-report | Emergency department presentation for skin infection or abscess | 114 | 0 | 36.7  (Mean) | 22 (19%) | NA |

**Supplementary table 24.** Studies evaluating the prevalence of mortality attributable to injecting-related infection

| **Study** | **Prevalence period** | **Method of infection assessment** | **Infection definition** | **Sample size** | **Number of males (%)** | **Mean or median age** | **Number of deaths due to infection (%)** | **95% confidence interval (CI)** |
| --- | --- | --- | --- | --- | --- | --- | --- | --- |
| Ersciou, 2013^86^ | 2011-2013 | Clinical record/ assessment | Death due to sepsis with methicillin susceptible Staphylococcus aureus | 249 | NR | NR | 12 (5%) | NA |
| Meisner, 2018^87^ | 2016-2017 | ICD codes | Death due to infective endocarditis | 669 | NR | NR | 3 (<1%) | NA |

**Reference list**

1. Alexander R, Agboola O, Costales V. Sociodemographic characteristics associated with hepatitis C among patients admitted for medically managed opioid withdrawal in east Tennessee. *Journal of Addictive Diseases* 2022; **40**(1): 92-5.

2. Ambekar A, Rao R, Mishra AK, Agrawal A. Type of opioids injected: does it matter? A multicentric cross-sectional study of people who inject drugs. *Drug Alcohol Rev* 2015; **34**(1): 97-104.

3. Asher AK, Zhong Y, Garfein RS, Cuevas-Mota J, Teshale E. Association of Self-Reported Abscess With High-Risk Injection-Related Behaviors Among Young Persons Who Inject Drugs. *Journal of the Association of Nurses in AIDS Care* 2019; **30**(2).

4. Aslam A, Rather S, Hussain A, Younus F, Saqib NU, Hassan I. Prevalence and Pattern of Dermatological Manifestations Among Substance Users Across Kashmir Valley in North India. *Indian Dermatol Online J* 2022; **13**(4): 457-65.

5. Axelsson A, Søholm H, Dalsgaard M, et al. Echocardiographic findings suggestive of infective endocarditis in asymptomatic Danish injection drug users attending urban injection facilities. *Am J Cardiol* 2014; **114**(1): 100-4.

6. Baltes A, Akhtar W, Birstler J, et al. Predictors of skin and soft tissue infections among sample of rural residents who inject drugs. *Harm Reduction Journal* 2020; **17**(1): 96.

7. Barocas JA, Hull SJ, Sosman JM, Sethi A, Fangman JJ, Westergaard R. Medical and legal consequences of ongoing drug use among young injection drug users infected with hepatitis C virus. J Gen Intern Med Abstracts of the 36th Annual Meeting of the Society of General Internal Medicine; 2013: S1-489.

8. Batisse A, Eiden C, Deheul S, Monzon E, Djezzar S, Peyriere H. Chemsex practice in France: An update in Addictovigilance data. *Fundam Clin Pharmacol* 2022; **36**(2): 397-404.

9. Benrubi LM, Silcox J, Hughto J, et al. Trends and correlates of abscess history among people who inject drugs in Massachusetts: A mixed methods exploration of experiences amidst a rapidly evolving drug supply. *Drug Alcohol Depend Rep* 2023; **8**: 100176.

10. Bicket MC, Park JN, Torrie A, Allen ST, Weir BW, Sherman SG. Factors associated with chronic pain and non-medical opioid use among people who inject drugs. *Addictive Behaviors* 2020; **102**: 106172.

11. Bonar EE, Rosenberg H. Using the health belief model to predict injecting drug users' intentions to employ harm reduction strategies. *Addictive Behaviors* 2011; **36**(11): 1038-44.

12. Bull-Otterson L, Huang YA, Zhu W, King H, Edlin BR, Hoover KW. Human Immunodeficiency Virus and Hepatitis C Virus Infection Testing Among Commercially Insured Persons Who Inject Drugs, United States, 2010-2017. *J Infect Dis* 2020; **222**(6): 940-7.

13. Calderon-Villarreal A, Abramovitz D, Avelar Portillo LJ, et al. Water, sanitation and hygiene insecurity predict abscess incidence among people who inject drugs in a binational US-Mexico metropolitan area: A longitudinal cohort study. *Int J Drug Policy* 2024; **129**: 104485.

14. Colledge-Frisby S, Jones N, Larney S, et al. The impact of opioid agonist treatment on hospitalisations for injecting-related diseases among an opioid dependent population: A retrospective data linkage study. *Drug Alcohol Depend* 2022; **236**: 109494.

15. Coull AF, Kyle RG, Hanson CL, Watterson AE. Risk factors for leg ulceration in people who inject drugs: A cross-sectional study. *J Clin Nurs* 2021; **30**(11-12): 1623-32.

16. Curtis SJ, Langham FJ, Tang MJ, et al. Hospitalisation with injection-related infections: Validation of diagnostic codes to monitor admission trends at a tertiary care hospital in Melbourne, Australia. *Drug Alcohol Rev* 2022; **41**(5): 1053-61.

17. Dahlman D, Håkansson A, Björkman P, Blomé MA, Kral AH. Correlates of Skin and Soft Tissue Infections in Injection Drug Users in a Syringe-Exchange Program in Malmö, Sweden. *Substance Use & Misuse* 2015; **50**(12): 1529-35.

18. Dahlman D, Håkansson A, Kral AH, Wenger L, Ball EL, Novak SP. Behavioral Characteristics and Injection Practices Associated with Skin and Soft Tissue Infections among People who Inject Drugs: A Community-Based Observational Study. *Substance Abuse* 2017; **38**(1): 105-12.

19. Dion K, Chiodo L, Whynott L, et al. Exploration of the unmet health care needs of people who inject drugs. *J Am Assoc Nurse Pract* 2020; **32**(1): 60-9.

20. Frank ND, Banta-Green CJ, Guthrie BL, et al. Emergency Room Utilization and Methamphetamine Overdose Symptoms Among Syringe Services Program Participants in Washington State. *Subst Use Misuse* 2024; **59**(7): 1012-9.

21. Frost MC, Williams EC, Kingston S, Banta-Green CJ. Interest in Getting Help to Reduce or Stop Substance Use Among Syringe Exchange Clients Who Use Opioids. *J Addict Med* 2018; **12**(6): 428-34.

22. Glick SN, Klein KS, Tinsley J, Golden MR. Increasing Heroin-Methamphetamine (Goofball) Use and Related Morbidity Among Seattle Area People Who Inject Drugs. *Am J Addict* 2021; **30**(2): 183-91.

23. Goncalves J, Madrid L, Donadille C, et al. Unsafe practices fostering cutaneous abscesses in people who inject substances : Results from the ANRS-OUTSIDER study. *Rev Epidemiol Sante Publique* 2023; **71**(5): 102142.

24. Heimer R, Khoshnood K, Crawford F, et al. Project CROSSROADS: Size Estimation, Risk Behavior Assessment, and Disease Prevalence in Populations at High Risk for HIV Infection in Lebanon 2015.

25. Horan JA, Van Hout MC. Mapping service user needs to inform a supervised injecting room location in Cork, Ireland. *Heroin Addiction and Related Clinical Problems* 2020; **22**(2): 1592-638.

26. Life MoHQo. A Respondent Driven Survey (RDS) among People Who Inject Drugs [PWIDs] in the Island of Mauritius, 2018.

27. Stafford J, Burns L. Australian Drug Trends 2010: Findings from the Illicit Drug Reporting System (IDRS). Australian Drug Trends Series, 2011.

28. Stafford J, Burns L. Australian Drug Trends 2011: Findings from the Illicit Drug Reporting System (IDRS). Australian Drug Trends Series, 2012.

29. Stafford J, Burns L. Australian Drug Trends 2012: Findings from the Illicit Drug Reporting System (IDRS). Australian Drug Trends Series, 2013.

30. Stafford J, Burns L. Australian Drug Trends 2013: Findings from the Illicit Drug Reporting System (IDRS). Australian Drug Trends Series, 2014.

31. Stafford J, Burns L. Australian Drug Trends 2014: Findings from the Illicit Drug Reporting System (IDRS). Australian Drug Trends Series, 2015.

32. Stafford J, Breen C. Australian Drug Trends 2015: Findings from the Illicit Drug Reporting System (IDRS). Australian Drug Trends Series, 2016.

33. Stafford J, Breen C. Australian Drug Trends 2016. Findings from the Illicit Drug Reporting System (IDRS). Australian Drug Trend Series. 2017.

34. Karlsson A, Burns L. Australian Drug Trends 2017: Findings from the Illicit Drug Reporting System (IDRS). Australian Drug Trends Series, 2018.

35. Peacock A, Gibbs D, Sutherland R, et al. Australian Drug Trends 2018: Key findings from the National Illicit Drug Reporting System (IDRS) Interviews, 2018.

36. Peacock A, Uporova J, Karlsson A, et al. Australian Drug Trends 2019: Key Findings from the National Illicit Drug Reporting System (IDRS) Interviews, 2019.

37. Peacock A, Uporova J, Karlsson A, et al. Australian Drug Trends 2020: Key Findings from the National Illicit Drug Reporting System (IDRS) Interviews, 2021.

38. Sutherland R, Uporova J, Chandrasena U, et al. Australian Drug Trends 2021: Key Findings from the National Illicit Drug Reporting System (IDRS) Interviews, 2021.

39. Sutherland R, Uporova J, King C, et al. Australian Drug Trends 2022: Key Findings from the National Illicit Drug Reporting System (IDRS) Interviews, 2022.

40. Sutherland R, Uporova J, King C, et al. Australian Drug Trends 2023: Key Findings from the National Illicit Drug Reporting System (IDRS) Interviews., 2023.

41. Sutherland R, Karlsson A, Uporova J, et al. Australian Drug Trends 2024: Key Findings from the National Illicit Drug Reporting System (IDRS) Interviews., 2024.

42. Ivan M, Rodgers C, Maher L, van Beek I. Reducing injecting-related injury and diseases in people who inject drugs: Results from a clinician-led brief intervention. *Aust Fam Physician* 2016; **45**(3): 129-33.

43. Johnson KE, Robinowitz N, Smith ME, Serio-Chapman C. Behavioral and demographic factors associated with acute and chronic wounds among clients of an injection drug syringe exchange program. 23rd Annual Meeting of the Wound Healing Society; SAWC Spring/WHS Joint Meeting; Silver (25th) Anniversary of the Wound Healing Society Wound Rep Reg; 2013: A28.

44. Jorgensen J, Dahlman D, Alanko Blome M, Janson H, Riesbeck K, Nilsson AC. Staphylococcus aureus carriage and prevalence of skin and soft tissue infections among people who inject drugs: a longitudinal study. *Sci Rep* 2024; **14**(1): 12919.

45. Lee WK, Ti L, Hayashi K, et al. Assisted injection among people who inject drugs in Thailand. *Substance Abuse Treatment, Prevention, and Policy* 2013; **8**(1): 32.

46. Li MY, Alba GA, Mitton J, Bearnot B. Care-engaged individuals with polysubstance use in Northeastern US are undertreated for methamphetamine use disorder: a retrospective cohort study. *Addiction Science & Clinical Practice* 2021; **16**(57).

47. Maloney S, Keenan E, Geoghegan N. What are the risk factors for soft tissue abscess development among injecting drug users? *Nursing Times* 2010; **106**(23): 21-4.

48. Marks LR, Durkin MJ, Ayres K, Ellis M. Drug preparation, injection-related infections, and harm reduction practices among a national sample of individuals entering treatment for opioid use disorder. *Harm Reduct J* 2024; **21**(1): 16.

49. McMahan VM, Kingston S, Newman A, Stekler JD, Glick SN, Banta-Green CJ. Interest in reducing methamphetamine and opioid use among syringe services program participants in Washington State. *Drug and Alcohol Dependence* 2020; **216**: 108243.

50. Megerian CE, Bair L, Smith J, et al. Health risks associated with smoking versus injecting fentanyl among people who use drugs in California. *Drug Alcohol Depend* 2024; **255**: 111053.

51. Mezaache S, Briand-Madrid L, Laporte V, Rojas Castro D, Carrieri P, Roux P. A syndemic examination of injecting drug use, incarceration and multiple drug-related harms in French opioid users. *International Journal of Prisoner Health* 2022; **18**(4): 417-28.

52. Morin KA, Prevost CR, Eibl JK, Franklyn MT, Moise AR, Marsh DC. A retrospective cohort study evaluating correlates of deep tissue infections among patients enrolled in opioid agonist treatment using administrative data in Ontario, Canada. *PLOS ONE* 2020; **15**(4): e0232191.

53. Public Health Scotland. The Needle Exchange Surveillance Initiative (NESI): Prevalence of blood-borne viruses and injecting risk behaviours among people who inject drugs attending injecting equipment provision services in Scotland, 2008 to 2020, 2022.

54. Public Health Scotland. The Needle Exchange Surveillance Initiative (NESI), 2023.

55. Noroozi M, Armoon B, Ghisvand H, et al. Prevalence and risk factors for injection site skin infections among people who inject drugs (PWID) in Tehran. *Journal of Cosmetic Dermatology* 2019; **18**(1): 258-62.

56. Ojha SP, Sigdel S, Meyer-Thompson H-G, Oechsler H, Verthein U. ‘South Asian cocktail’ - the concurrent use of opioids, benzodiazepines and antihistamines among injecting drug users in Nepal and associations with HIV risk behaviour. *Harm Reduction Journal* 2014; **11**(1): 17.

57. Ozga JE, Syvertsen JL, Zweifler JA, Pollini RA. A community-based study of abscess self-treatment and barriers to medical care among people who inject drugs in the United States. *Health & Social Care in the Community* 2022; **30**(5): 1798-808.

58. Panda S, Roy T, Pahari S, et al. Alarming epidemics of human immunodeficiency virus and hepatitis C virus among injection drug users in the northwestern bordering state of Punjab, India: prevalence and correlates. *Int J STD AIDS* 2014; **25**(8): 596-606.

59. Perri MM, Culbert GJ, Mayer S, Jimenez AD, Yawanis J. Prevention of Skin and Soft Tissue Infections in People Who Inject Drugs. J Addict Med; 2021: E47.

60. Peyriere H, Eiden C, Micallef J, Lapeyre-Mestre M, Faillie JL, Blayac JP. Slow-release oral morphine sulfate abuse: results of the postmarketing surveillance systems for psychoactive prescription drug abuse in France. *Eur Addict Res* 2013; **19**(5): 235-44.

61. Roux P, Donadille C, Magen C, et al. Implementation and evaluation of an educational intervention for safer injection in people who inject drugs in Europe: a multi-country mixed-methods study. *International Journal of Drug Policy* 2021; **87**: 102992.

62. Roux P, Jauffret-Roustide M, Donadille C, et al. Impact of drug consumption rooms on non-fatal overdoses, abscesses and emergency department visits in people who inject drugs in France: results from the COSINUS cohort. *International Journal of Epidemiology* 2022; **52**(2): 562-76.

63. Schneider KE, White RH, Rouhani S, Tomko C, Nestadt DF, Sherman SG. Self and professional treatment of skin and soft tissue infections among women who inject drugs: Implications for wound care provision to prevent endocarditis. *Drug and Alcohol Dependence Reports* 2022; **3**: 100057.

64. Smith ME, Robinowitz N, Chaulk P, Johnson KE. High rates of abscesses and chronic wounds in community-recruited injection drug users and associated risk factors. *J Addict Med* 2015; **9**(2): 87-93.

65. Srivastava P, Modi V, Lier A. Evaluation of Infectious Diseases Complications in US Veterans with Opioid Use Disorder, a Single Site Experience. Open Forum Infectious Diseases; 2023: ofad500.2126.

66. Summers PJ, Hellman JL, MacLean MR, Rees VW, Wilkes MS. Negative experiences of pain and withdrawal create barriers to abscess care for people who inject heroin. A mixed methods analysis. *Drug and Alcohol Dependence* 2018; **190**: 200-8.

67. Syvertsen J. Social context and high risk injection drug practices in Nyanza, Kenya: Implications for further research & interventions. 2014.

68. Taylor H, Curado A, Tavares J, Oliveira M, Gautier D, Maria JS. Prospective client survey and participatory process ahead of opening a mobile drug consumption room in Lisbon. *Harm Reduction Journal* 2019; **16**(1): 49.

69. Public Health England NIS. Unlinked Anonymous Monitoring Survey of People Who Inject Drugs: Data tables., 2017.

70. Public Health England NIS. Unlinked Anonymous Monitoring Survey of People Who Inject Drugs: Data tables., 2018.

71. Public Health England NIS. Unlinked Anonymous Monitoring Survey of People Who Inject Drugs: Data tables., 2019.

72. Public Health England NIS. Unlinked Anonymous Monitoring Survey of People Who Inject Drugs: Data tables., 2020.

73. Public Health England NIS. Unlinked Anonymous Monitoring Survey of People Who Inject Drugs: Data tables., 2021.

74. Wright T, Hope V, Ciccarone D, Lewer D, Scott J, Harris M. Prevalence and severity of abscesses and cellulitis, and their associations with other health outcomes, in a community-based study of people who inject drugs in London, UK. *PLoS One* 2020; **15**(7): e0235350.

75. Wurcel AG, Burke D, Skeer M, et al. Sex work, injection drug use, and abscesses: Associations in women, but not men. *Drug Alcohol Depend* 2018; **185**: 293-7.

76. Yen Y-F, Chou P, Lin Y-S, Deng C-Y. Factors associated with health-related quality of life among injection drug users at methadone clinics in Taipei, Taiwan. *Journal of the Chinese Medical Association* 2015; **78**(5): 292-8.

77. Robert Koch-Institut. Abschlussbericht der Studie „Drogen und chronischen Infektionskrankheiten in Deutschland“ (DRUCK-Studie). Berlin: Robert Koch-Institut, 2016.

78. Bonacci RA, Moorman AC, Bixler D, et al. Prevention and Care Opportunities for People Who Inject Drugs in an HIV Outbreak — Kanawha County, West Virginia, 2019–2021. *J Gen Intern Med* 2022.

79. Dahlman D, Berge J, Björkman P, Nilsson AC, Håkansson A. Both localized and systemic bacterial infections are predicted by injection drug use: A prospective follow-up study in Swedish criminal justice clients. *PLOS ONE* 2018; **13**(5): e0196944.

80. Figgatt MC, Schranz AJ, Jackson BE, et al. Mortality associated with bacterial and fungal infections and overdose among people with drug use diagnoses. *Ann Epidemiol* 2023; **87**.

81. Nambiar D, Stoové M, Hickman M, Dietze P. A prospective cohort study of hospital separations among people who inject drugs in Australia: 2008–2013. *BMJ Open* 2017; **7**: e014854.

82. Vanichseni S, Martin M, P S, et al. High Mortality Among Non–HIV-Infected People Who Inject Drugs in Bangkok, Thailand, 2005–2012. *American Journal of Public Health* 2015; **105**(6).

83. Wang L, Volkow ND, Berger NA, Davis PB, Kaelber DC, Xu R. Association of COVID-19 with endocarditis in patients with cocaine or opioid use disorders in the US. *Mol Psychiatry* 2023; **28**(2): 543-52.

84. Evans ME, Person M, Reilley B, al. e. Trends in Indicators of Injection Drug Use, Indian Health Service, 2010-2014: A Study of Health Care Encounter Data. *Public Health Reports* 2020; **135**(4): 461-71.

85. Wheeler A, Valerio H, Cunningham EB, et al. Prevalence and factors associated with hospitalisation for bacterial skin infections among people who inject drugs: The ETHOS Engage Study. *Drug and Alcohol Dependence* 2022; **237**: 109543.

86. Erscoiu S, Popa I, Oncel D, Burcoş O, Pătru M, Ceauşu E. Causes of mortality among new HIV-infected intravenous drug users in a clinical hospital in Bucharest. *BMC Infectious Diseases* 2013; **13**(1): O6.

87. Meisner J, Koenig H, Tebas P. Synergizing Infectious Diseases and Substance Use Treatment to Improve the Outcomes of Endocarditis in People Who Inject Drugs at a Large Academic Hospital. *Open Forum Infectious Diseases* 2018; **5**: S306.
